# Dual Fatty Acid Elongase Complex Interactions in Arabidopsis

**DOI:** 10.1371/journal.pone.0160631

**Published:** 2016-09-01

**Authors:** Céline Morineau, Lionel Gissot, Yannick Bellec, Kian Hematy, Frédérique Tellier, Charlotte Renne, Richard Haslam, Frédéric Beaudoin, Johnathan Napier, Jean-Denis Faure

**Affiliations:** 1 Institut Jean-Pierre Bourgin, INRA, AgroParisTech, CNRS, Université Paris-Saclay, Versailles, France; 2 Univ Paris-Sud, Université Paris-Saclay, Orsay, France; 3 Department of Biological Chemistry and Crop Protection, Rothamsted Research, Harpenden, Herts, United Kingdom; Louisiana State University Health Sciences Center, UNITED STATES

## Abstract

Very long chain fatty acids (VLCFAs) are involved in plant development and particularly in several cellular processes such as membrane trafficking, cell division and cell differentiation. However, the precise role of VLCFAs in these different cellular processes is still poorly understood in plants. In order to identify new factors associated with the biosynthesis or function of VLCFAs, a yeast multicopy suppressor screen was carried out in a yeast mutant strain defective for fatty acid elongation. Loss of function of the elongase 3 hydroxyacyl-CoA dehydratase *PHS1* in yeast and *PASTICCINO2* in plants prevents growth and induces cytokinesis defects. *PROTEIN TYROSIN PHOSPHATASE-LIKE (PTPLA)* previously characterized as an inactive dehydratase was able to restore yeast *phs1* growth and VLCFAs elongation but not the plant *pas2-1* defects. PTPLA interacted with elongase subunits in the Endoplasmic Reticulum (ER) and its absence induced the accumulation of 3-hydroxyacyl-CoA as expected from a dehydratase involved in fatty acid (FA) elongation. However, loss of PTPLA function increased VLCFA levels, an effect that was dependent on the presence of PAS2 indicating that PTPLA activity repressed FA elongation. The two dehydratases have specific expression profiles in the root with *PAS2*, mostly restricted to the endodermis, while *PTPLA* was confined in the vascular tissue and pericycle cells. Comparative ectopic expression of PTPLA and PAS2 in their respective domains confirmed the existence of two independent elongase complexes based on PAS2 or PTPLA dehydratase that are functionally interacting.

## Introduction

Very long chain fatty acids (VLCFAs) are fatty acids (FA) with an acyl chain longer than 18 carbons. They are components of a large variety of plant lipids like the membrane lipids phospholipids and sphingolipids, the storage lipids triacylglycerol and the hydrophobic lipid barrier comprising cuticular waxes and suberin.

VLCFAs are elongated in the endoplasmic reticulum (ER) by the elongase complex that sequentially adds two carbons to long chain acyl-CoAs (16 or 18 carbons) originating from *de novo* synthesis in the plastids. The elongase complex includes four enzymes starting with, the 3-keto-acyl-CoA synthase (KCS) that condensates the acyl-CoA with a malonyl-CoA to form a 3-ketoacyl-CoA intermediate that is in a second step reduced by the β-ketoacyl-CoA reductase (KCR) in 3-hydroxyacyl-CoA. The 3-hydroxyacyl-CoA dehydratase (HCD) then dehydrates the 3-hydroxyacyl-CoA in trans-2,3-enoyl-CoA that is finally reduced by the fourth enzyme, the trans-2,3-enoyl reductase (ECR). The acyl-CoA elongated by two carbons can re-enter an elongation cycle to eventually produce VLCFAs ranging from C18 to C32 in Arabidopsis.

In Arabidopsis, 21 *FAE-like/KCS* genes grouped in 8 distinct subclasses [1] encode the condensing component of the elongase complex. The different KCS are characterized by their substrate (acyl chain length) and tissue specificities [1–3]. The three other elongase subunits show a much lower gene diversity in Arabidopsis. Two genes, *KCR1* and *KCR2* are homologous to yeast *KCR YBR159*. However, only KCR1 is able to restore elongase activity in *ybr159* yeast mutant [4]. Similarly, Arabidopsis genome presents two genes with similarity to yeast 3-hydroxyacyl-CoA dehydratase *PHS1*, *PASTICCINO2* (*PAS2*) and *PROTEIN TYROSINE PHOSPHATASE-LIKE* (*PTPLA*) [5,6]. Like *KCR1* with *ybr159*, only *PAS2* was able to complement null yeast *phs1* mutant [5]. Finally, ECR is encoded by *CER10* that complements the *tsc13* yeast mutant [7,8]. Beyond these models model species for fungi and plants, HCD-encoding genes are important for human and dog health or basidiomycete survival [9–11].

VLCFAs are essential lipids since all the mutations in yeast and plants preventing acyl-CoA elongation result in lethality [5,12]. *A*. *thaliana kcr1* and *pas2* loss of function mutants led to global decrease of the VLCFA in the different lipid pools and to embryo lethality [4,5]. Silencing of the tobacco ECR in leaves leads to necrotic lesions and epidermal cell ablation [13]. Cell death could also be observed in plants with ectopic expression of seed specific KCS *FAE1* in the epidermis indicating that the nature and amount of VLCFA are important for cell viability [14]. Likewise, enhancement of VLCFA levels altered plant development as illustrated by the KCS FAE1 or yeast PHS1 overexpression in Arabidopsis [5,15]. VLCFA were directly involved in cell differentiation and lateral root organogenesis by promoting polar auxin transport in the *pas1* mutant [16]. Sphingolipids are most likely involved in polar auxin transport since ceramide synthase mutants *loh1/loh3* also showed a reduced auxin-dependent lateral root initiation [17]. Defective development associated with unbalanced VLCFA/LCFA ratio was often observed with some membrane defects [8,15]. Reduced VLCFA elongation impaired cell elongation and division especially membrane trafficking during cell plate formation [5,18], but also altered Fts-Z assembly during plastid division [19]. Specific depletion of VLCFA in sphingolipids induced also membrane trafficking and cytokinesis defects that could be related to enhanced stability in endosomal transient interactions (Markham et al., 2011, Molino et al., 2014). *In vitro* experiments directly demonstrated the importance of acyl chain length of the sphingolipid glucosylceramide in liposome fusion [20].

Apart from being structural components of membrane lipids, VLCFAs have also other functions in plant development. VLCFA are essential components of cuticular and epicuticular waxes that were responsible, when missing, for post-genital organ fusion [21–23]. Interestingly, reduced FA elongation by mutation of cytosolic acetyl carboxylase PAS3 or the VLCFA dehydratase PAS2 was correlated with cytokinin hypersensitivity and cell proliferation [21,24] and recently VLCFA were described as potential non-cell autonomous regulators of plant development by repressing cytokinin synthesis [25].

In yeast, partial inactivation of FA elongation led to biochemical and cytokinesis defects similar to those observed in Arabidopsis [6,18,26,27]. Yeast *phs1* and Arabidopsis *pas2-1* mutants showed reduced Acyl-CoA elongation, associated with 3-hydroxyacyl-CoA accumulation, and an increase in free sphingoid base like phytosphingosine (PHS) [5,28]. To identify new components of FA elongation, we took advantage of these similarities to carry out a suppressor screen of a leaky *phs1* strain (*Tet-PHS1)* with an *A*. *thaliana* cDNA library. We identified PTPLA as a suppressor of the *Tet-PHS1* yeast strain that was able to restore both the *Tet-PHS1* yeast growth and the FA elongation defects. PTPLA was however not able to rescue the developmental defects of the Arabidopsis *pas2-1* mutant but could further enhance FA elongation in presence of an active PAS2. The loss of *ptpla* function was characterized by 3-hydroxyacyl-CoA accumulation as expected for a FA elongase dehydratase but surprisingly also led to the accumulation of VLCFA. The non-overlapping expression pattern between the two dehydratases led us to propose the existence of a second elongase complex associated with PTPLA that was involved in repressing the activity of the major elongase complex comprising PAS2 dehydratase. A plant like Arabidopsis would thus have two different elongase complexes functionally interacting in adjacent cell tissues.

## Results

### Arabidopsis PTPLA rescues the *S*. *cerevisiae acyl-CoA dehydratase Tet-PHS1* mutant

**Fig 1:**
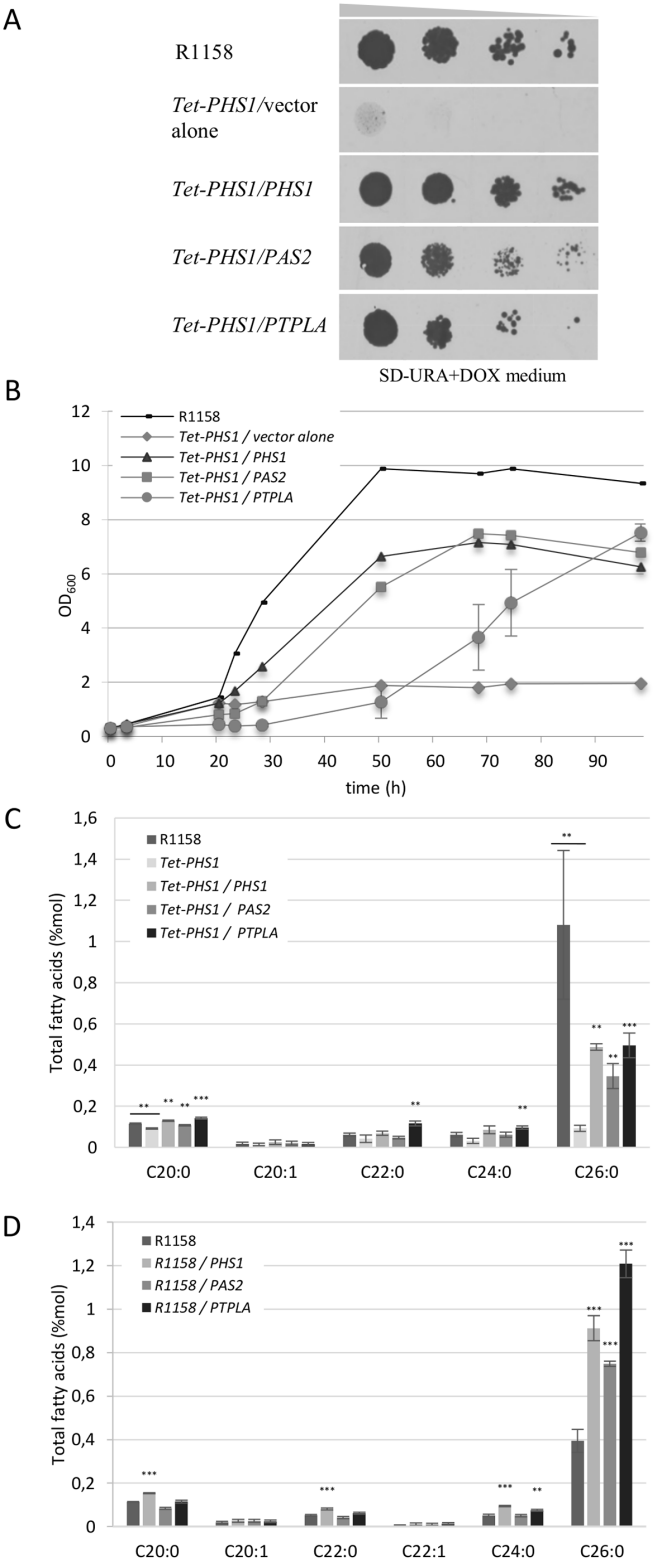
*A*. *thaliana PTPLA* complements inducible yeast *phs1* mutant. (A) PTPLA expression restores growth of *Tet-PHS1* in presence of DOX. *Tet-PHS1* was transformed with yeast expression vector pFL61 alone or with yeast *PHS1*, Arabidopsis *PAS2* or *PTPLA*. R1158 is the wild type control strain. (B) Growth kinetic of *Tet-PHS1* strain expressing PHS1, PAS2 and PTPLA in presence of *DOX*. Three independent PTPLA expressing clones were analyzed and the mean (+/- sd) is shown. (C-D) Fatty acid content of PTPLA expressing yeasts. PTPLA expression induces fatty acid elongation in yeast *Tet-PHS1* in presence of *DOX* (C) and in wild type strain (D). The graph shows FAMES analysis from n = 5–12 and n = 9–15 independent clones for respectively (C) and (D). Data shows means (+/- se). Significant differences between *Tet-PHS1* (C) or the wild-type (D) and overexpressing strains were determined using the Wilcoxon-test: *p<0,05, **p<0,01, ***p < 0.001.

To identify new genes able to suppress VLCFA depletion defects, yeast *Tet-PHS1* mutant was transformed with an Arabidopsis cDNA library. Since null *phs1* mutation is lethal, an inducible strain was used *(Tet-PHS1)* (S1 Fig). A total of 698 clones growing on selective medium were selected, sequenced and confirmed in a second screen. Two cDNAs were identified as strong suppressor of *Tet-PHS1*. As expected, *PHS1* ortholog *PAS2* (AT5G10480) corresponded to the majority of the yeast clones (457 clones) but a second related cDNA, *PTPLA* (AT5G59770) was also identified in 35 clones (Fig 1A). PTPLA is closely related to PAS2 and PHS1 (respectively 32% and 35% of identity) (S2 Fig). The PHS1 protein has six transmembrane domains, a C-terminal retention signal to the ER and a dehydratase domain that has been shown to have three essential amino acids necessary for PHS1 dehydratase activity [29]. PTPLA and PAS2 proteins showed respectively five and four putative transmembrane domains (S2 Fig). Both proteins presented also a retention signal to the ER (KXKXX or KKXX) and the three conserved and essential amino acids required for dehydratase activity (S2 Fig). A previous study demonstrated that PAS2 was able to complement a null-*phs1* mutant while PTPLA could not, suggesting different activity between the two proteins [5]. The absence of complementation of null-phs1Δ strain by PTPLA was confirmed with the clones isolated in TET-PHS1 screen (S1B Fig). The phenotype of the PTPLA complementation of *Tet-PHS1* mutant was thus more carefully evaluated. First, PTPLA was able to restore the growth of the *Tet-PHS1* strain in presence of doxycycline (*Tet-PHS1*+DOX) to levels comparable to *Tet-PHS1*+DOX strain transformed with *PHS1* or *PAS2* cDNA albeit the kinetics of growth was slower (Fig 1B). The absence of the dehydratase PHS1 blocked fatty acid elongation and led to reduced VLCFA levels in yeast. As a corollary, phytosphingosine (PHS) level was enhanced since VLCFA are required for sphingolipids synthesis. *PTPLA* expression in *Tet-PHS1*+DOX was able to reduce PHS levels (S3 Fig), and induce VLCFA elongation to levels similar to what was observed for *Tet-PHS1*+DOX expressing PHS1 or PAS2 (Fig 1C and S4A Fig). For example, C26 amounts were increased by 5.4-fold in *Tet-PHS1*+DOX expressing *PTPLA* that is comparable to the ratio observed in *Tet-PHS1*+DOX expressing *PHS1* or *PAS2* (respectively of 5.2 and 3.7). Interestingly, a similar increase of VLCFA amounts was observed in wild-type R1158 yeast strain expressing *PTPLA* with more than a two-fold increase that was comparable to the effect of *PHS1* and *PAS2* expression in wild-type R1158 yeast strain (Fig 1D and S4B Fig). Finally, the hallmark of acylCoA dehydratase deficiency in yeast and in plants is the accumulation of the precursors, the 3-hydroxyacyl-CoAs [5,6]. The expression of PTPLA in *Tet-PHS1*+DOX strain led to the reduction of 3-hydroxy C20-CoA accumulation to the same extent as what was observed for *Tet-PHS1*+DOX strain expressing PHS1 (S5 Fig). All these data indicate that PTPLA was able to rescue PHS1 deficiency in the *Tet-PHS1*+DOX strain. The fact that PTPLA could not complement null-*phs1* strain would suggest that a minimal endogenous dehydratase activity was necessary for PTPLA suppressing activity.

### II PTPLA does not complement *A*. *thaliana pas2-1* mutant but enhances VLCFA levels

**Fig 2:**
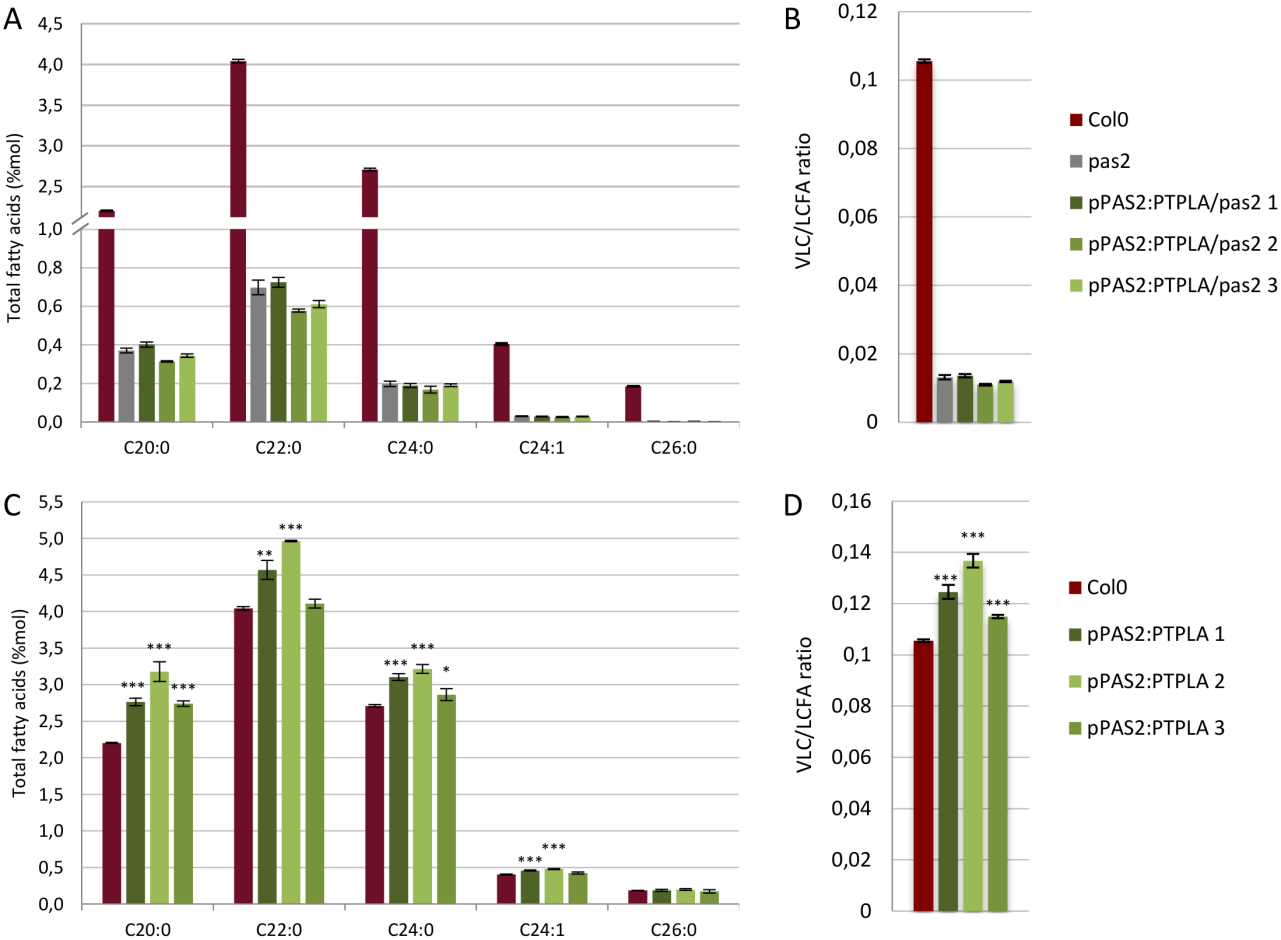
PTPLA expression enhances VLCFA contents in *A*. *thaliana*. Relative fatty acid content of *pas2-1* mutant (A and B) and Col0 (C and D) lines expressing 3 *pPAS2*:*PTPLA* independent constructs or the control *pPAS2*:*PAS2*. FAMES analysis were performed on roots of 14 days-old seedling (n = 3). (B and D) VLC/LCFA ratio shows means (+/- sd) of the ratio between very long chain (C20 to C26) and the long fatty acids (C16 and C18). Significant differences between *pas2-1* (A) or Col0 (B) and overexpressing lines were determined using the student’s t-test: *p<0,05, **p<0,01, ***p < 0.001.

The lack of complementation of a null allele of yeast *phs1* could be caused by some plant specific determinants of PTPLA activity. We thus evaluated if PTPLA was able to complement *Arabidopsis thaliana pas2-1* mutant, which has a reduced dehydratase activity associated with strong developmental defects [5,18]. The disruption of VLCFA elongation in *pas2-1* mutant induces cell proliferation and abnormal cytokinesis leading to defective differentiation in the apical part and shorter primary root [18]. These developmental defects were linked with reduced VLCFA levels in triglycerides, waxes, sphingolipids and phospholipids [5,18]. Moreover, the complete loss of PAS2 function is embryo lethal [5]. *PTPLA* was thus expressed in the heterozygous *pas2-1*/+ plant under the control of either the 35S or *PAS2* promoters. Segregation of *pas2-1/+* plants carrying either *35S*:*PTPLA* or *pPAS2*:*PTPLA* constructs showed around 25% *pas2-1/pas2-1* mutants in T2 progeny indicating an absence of complementation of *pas2-1* phenotype whereas *pPAS2*:*PAS2* totally rescue *pas2-1* phenotype (S1 Table). Correct *PTPLA* or *PAS2* expression and tissular localisation were controlled by quantitative RT-PCR (qRT-PCR) and by the observation of GFP-PAS2 or GFP-PTPLA fluorescence (S6 Fig). *PTPLA* expression under *pPAS2* promoter did not increase VLCFA levels in *pas2-1* mutant (Fig 2A and 2B) while *pPAS2*:*PAS2*, completely rescued VLCFA deficiency (S7D Fig). Interestingly, *pPAS2*:*PTPLA* or *p35S*:*PTPLA* expression in wild-type led to a significant increase of VLCFA content as seen in yeast (Fig 2C and 2D and S7B Fig) even if no clear overexpression of *PTPLA* transcripts could be observed (S6G Fig). These data suggest that ectopic expression of PTPLA was sufficient for enhancing VLCFA synthesis in wild-type context but was not able to functionally replace defective PAS2 dehydratase.

### III *PTPLA* is specifically expressed in root vascular tissues

**Fig 3:**
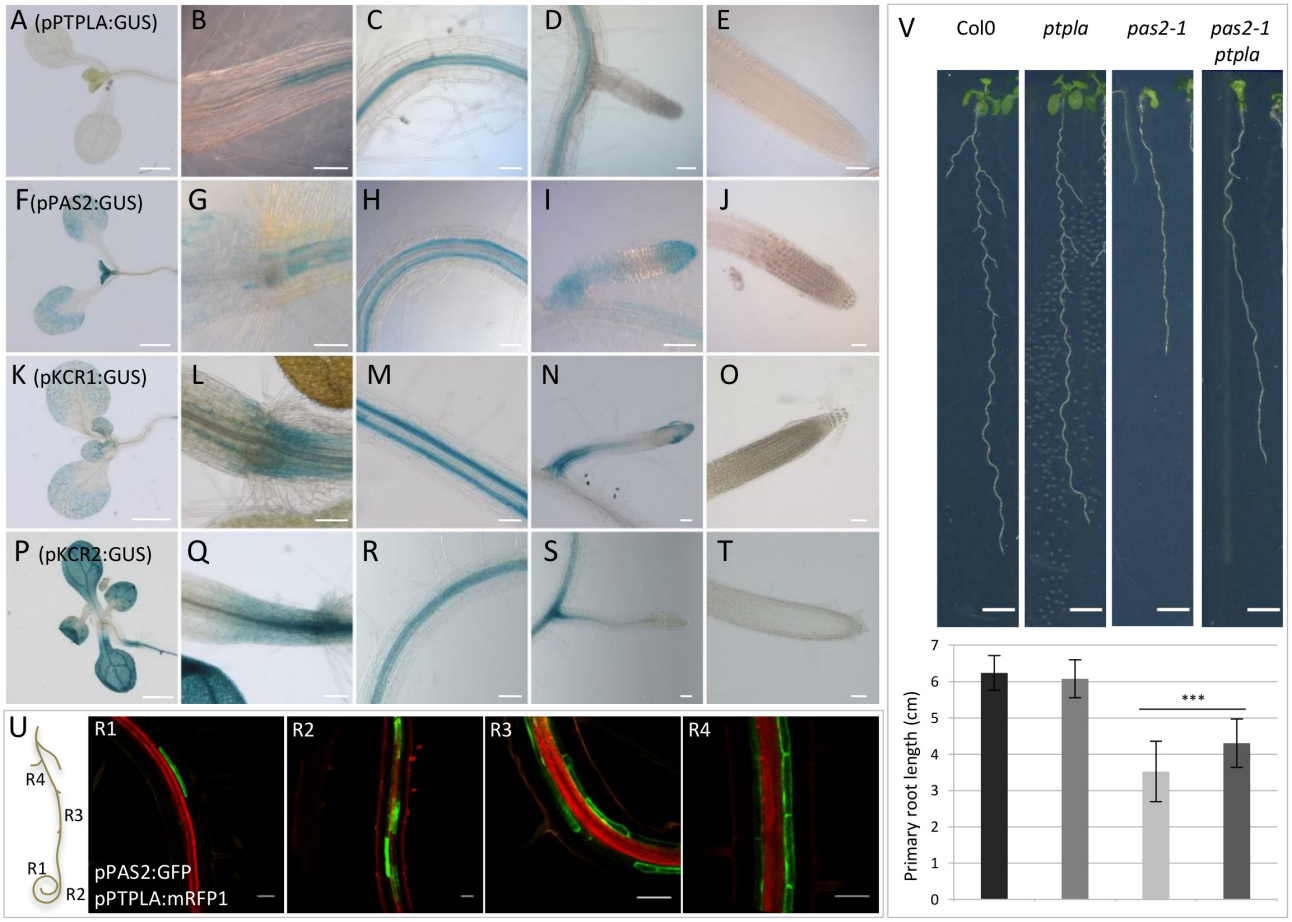
*PTPLA* is expressed during root development. (A-T) Expression of GUS constructs under the control of *pPTPLA* (A-E), *pPAS2* (F-J), *pKCR1* (K-O) or *pKCR2* promoters (P-T) in 14 days-old Arabidopsis seedlings. GUS staining was observed in different organs: seedling apical part (A, F, K, P), hypocotyl/root transition (B, G, L,Q), root hair transition zone of the primary root (C, H, M, R), secondary root (D, I, N, S) and primary root tip (E, J, O, T). Scale: 50 μm (except pictures A, F, K, P: 2mm). n = 18. (U). Coexpression of *pPTPLA*:*mRFP1* and *pPAS2*:*GFP* in *A*. *thaliana* primary root. Images were taken from the tip (R1), the root hair initiation zone (R2), the lateral root initiation zone and lateral root emergence zone (R4). Scale: 50μm. (V) Effect of *ptpla* mutation of primary root length. Vertically grown 10 days-old seedling (top) and the corresponding primary root length (bottom). n = 43–57. Scale: 500μm. Significant differences were determined using the student’s t-test: *p<0,05, **p<0,01, ***p < 0.001.

To better understand the difference between PTPLA and PAS2 in fatty acid elongation, expression patterns of several *pPAS2* and *pPTPLA* reporter fusions were compared in stable transgenic lines (promoter size and number of lines are described in methods). *pPTPLA*:*GUS* staining was specifically localized in mature primary and secondary roots and restricted to the central cylinder (Fig 3A–3E). *pPAS2*:*GUS* staining was also present in mature roots and secondary roots but also expressed in the epidermis of cotyledons and leaves (Fig 3F–3J and S6F Fig) as previously described [25]. Interestingly, in mature primary roots *pPTPLA*:*GUS* appeared to be restricted to vascular tissue while *pPAS2*:*GUS* expression profile was specific to the endodermis (Fig 3C and 3H). Stable co-expression of *pPTPLA*:*mRFP1* and *pPAS2*:*GFP* in *Arabidopsis thaliana* showed clearly that the two genes have non-overlapping expression profiles (Fig 3U). *pPTPLA*:*mRFP1* showed a continuous expression in vascular tissue from the differentiation zone of the meristem to the root-hypocotyl junction while *pPAS2*:*GFP* was only expressed in the endodermis, first in few cells leading to a patchy staining and eventually in every endodermal cell. The non-overlapping and specific expression patterns of *pPTPLA* and *pPAS2* suggested a spatial regulation of VLCFA synthesis. The condensing enzyme KCS, the first enzyme of the elongase complex is encoded by a large gene family which presents a different expression profiles [1,30]. Several KCS transcripts are expressed in the roots [1,31] and at least KCS2 and KCS20 were specifically expressed in root endodermis [32]. In a similar way to the dehydratase, the 3-ketoacyl-CoA reductase is encoded by two genes (KCR1 and KCR2) but only KCR1 was able to complement yeast *ybr159* mutation [4]. Our intention was to examine if *KCR1* and *KCR2* genes have similar expression profiles to *PAS2* and *PTPLA*. Analysis of GUS expression in stable transgenic lines expressing *pKCR1*:*GUS* and *pKCR2*:*GUS* showed different expression patterns in the root that matched those of *pPAS2*:*GUS* and *pPTPLA*:*GUS* respectively. *KCR1* and *PAS2* promoters showed expression in cotyledons, leaves and a specific staining in the endodermis of the roots (Fig 3F–3J and 3K–3O). *pPTPLA*:*GUS* and *pKCR2*:*GUS* stained vascular tissues of mature primary and secondary root (Fig 3A–3E and 3P–3T). Contrary to the *PTPLA* promoter, the *KCR2* promoter was also expressed in cotyledons, leaves and in the meristem of secondary roots (Fig 3P–3T).

We then checked whether restricted *PTPLA* expression was associated with root development. The *ptpla* mutant was characterized by a T-DNA insertion in the seventh intron (S8A and S8B Fig) leading to at least 90% reduction of a truncated *PTPLA* mRNA (S6G and S8C Figs). Contrary to *pas2-1*, which showed shorter primary roots compared to wild type, the length of the primary root as well as the number of lateral roots were not altered in the *ptpla* mutant (Fig 3V and S9 Fig). However, the primary root was slightly longer in the double *pas2-1/ptpla* mutant compared to the single *pas2-1* mutant suggesting that the absence of *PTPLA* partially rescued *pas2-1* root growth. The phenotype was observed at two developmental stages in two independent experiments in 10 (Fig 3V) and 14 day-old seedlings (S9A Fig) respectively.

### IV PTPLA associates with the elongase complex in the ER

**Fig 4:**
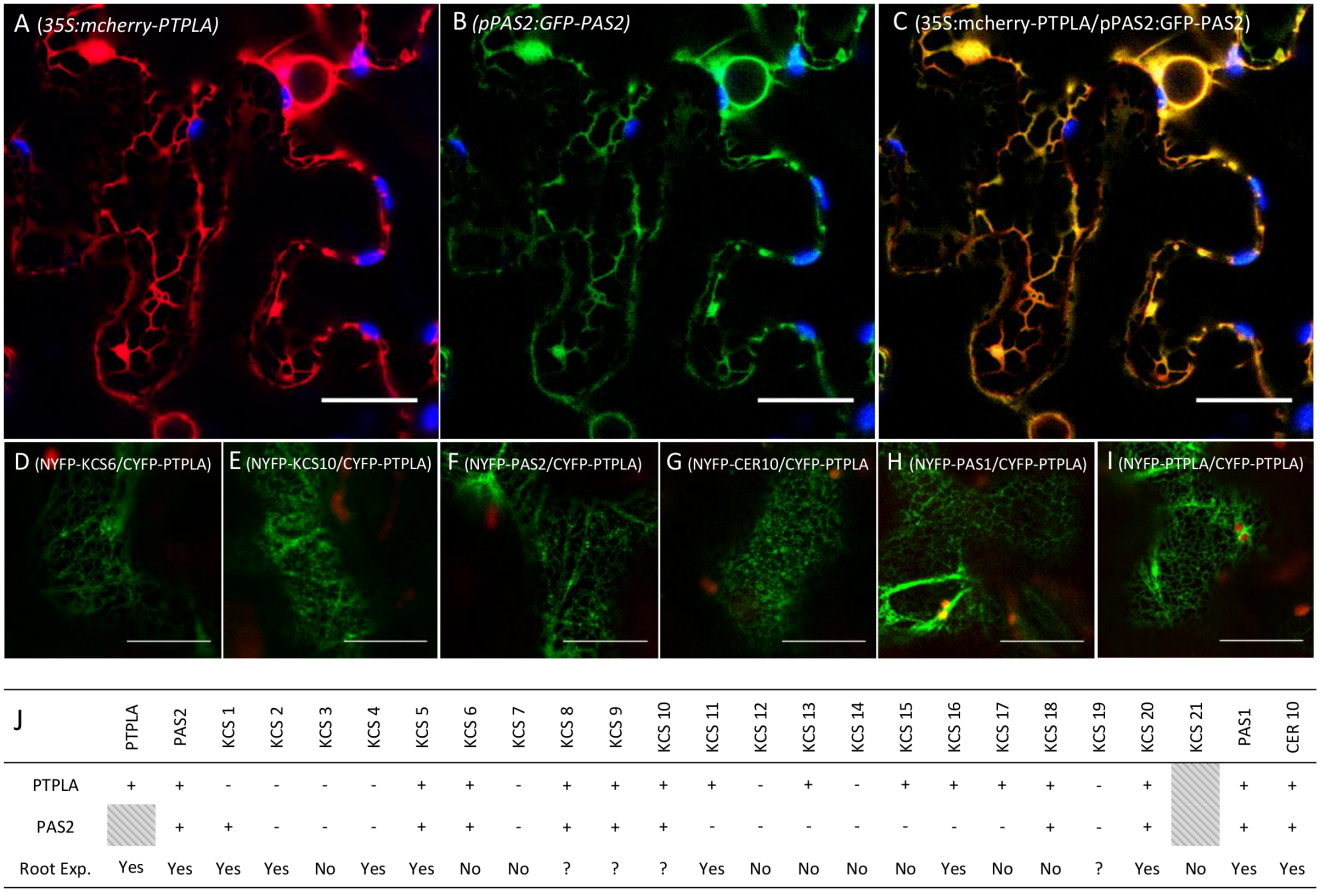
PTPLA interacts with the elongase complex subunits in the ER. (A-C) Subcellular distribution in *N*.*benthamiana* epidermal cells of *35S*:*mcherry-PTPLA* (A) and *pPAS2*:*GFP-PAS2* (B). Merged channels showed colocalization *(C)*. Chloroplast autofluorescence is shown in blue. Scale: 50μm. (D-I). BiFC assays between PTPLA and subunits of the elongase complex in the ER. Scale: 25μm. (J) Results of BiFC assays between PTPLA and several enzymes of the elongase complex. The plus sign (+) indicates an interaction, the minus sign (-) no interaction. Root Exp.: Root expression of the proteins or genes when is known [1,4,5,31] is indicated by “Yes”. The question mark indicates unclear information about the KCS expression. “No” indicates no or unknown expression of the protein in the root.

In plants, the elongase complex is localized in the ER [1,4,13]. Subcellular localization of PTPLA was first characterized with the transient expression of *35S*:*mCherry-PTPLA* constructs in *Nicotiana benthamiana*. The subcellular distribution of mCherry-PTPLA showed the characteristic ER network which was confirmed by its colocalization with ER localized GFP-PAS2 fusion (Fig 4A–4C). To investigate whether PTPLA was directly associated with enzymes of the elongase complex, *in vivo* protein-protein interaction assays were carried out by Bimolecular Fluorescence Complementation (BiFC) experiments. PTPLA and different subunits of the elongase complex were fused with C_YFP_- or N_YFP_- at the N terminal of the proteins of interest to prevent potential interference with ER retention signal located at the C-terminal end and transiently expressed in *Nicotiana benthamiana* leaves (Fig 4D–4I). The different combinations of proteins are summarized in Fig 4D–4I. Direct interaction of PTPLA with core elongase subunits KSC6, KCS10 and the reductase CER10 could be observed in the ER (Fig 4D–4I) but also with, the potential elongase chaperone, the immunophilin PASTICCINO1 [16]. All interactions observed by confocal microscopy are shown in S10 Fig. PTPLA and PAS2 interacted with the same KCS like KCS5, 6, 8, 9, 10 and 18 but also showed specific association with KCS1 for PAS2 or KCS 11, 13, 15 and 17 for PTPLA (Fig 4J). No interaction could be observed with 6 KCS including KCS2 for both PTPLA and PAS2 suggesting that this assay might be too stringent for evaluating some protein elongase associations. Most of the elongase subunits interacting with PTPLA and with a known expression profile were expressed in the root. Interestingly, PTPLA could also interact with itself and with PAS2 suggesting that acyl-CoA dehydratase could form homo- or hetero-dimers within the elongase complex in p35S:PTPLA or pPAS2:PTPLA transgenic lines. Interaction assay with KCR1 or KCR2 could unfortunately not be carried out since KCR fusion proteins were not correctly expressed with this transient system. In vivo split-luciferase assays confirmed that PTPLA homo or hetero-dimerization with PAS2 were as strong as the interaction with another complex subunit like CER10 indicating a direct involvement of PTPLA in the elongase complex (S11 Fig).

### V *ptpla* mutant accumulates hydroxyacyl-CoA and VLCFA

**Fig 5:**
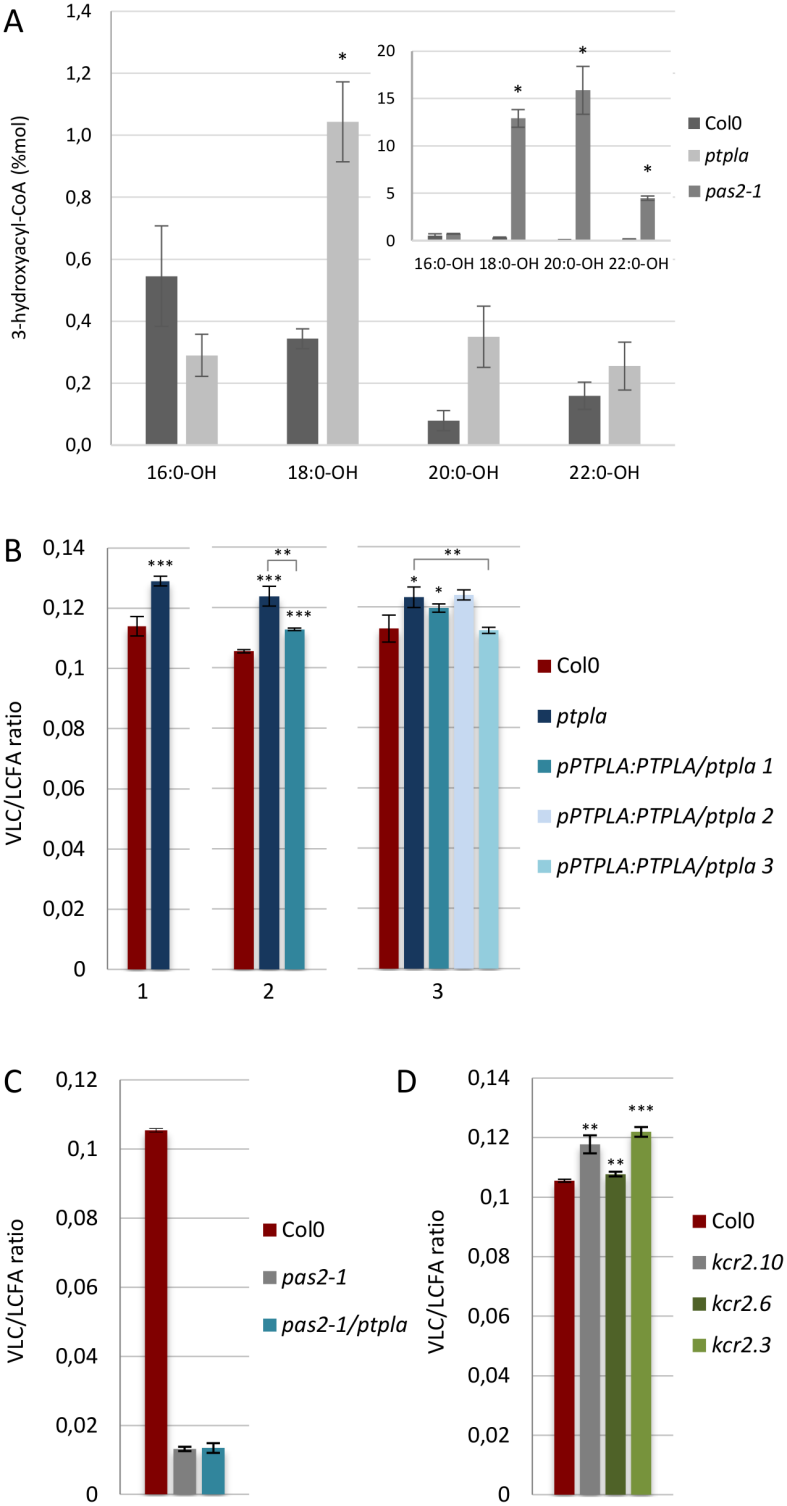
PTPLA is involved in very long chain fatty acids elongation. (A) 3-hydroxy-acyl-CoA profile of *pas2-1* and *ptpla* mutant roots compared to wild type. n = 4. Significant differences were determined using the Wilcoxon-test: *p<0,05, **p<0,01, ***p < 0.001. (B) Three independent experiments showing the VLC/LCFA ratio in *pas2-1* and *ptpla* mutant roots compared to wild type. Three independent *ptpla* mutant lines expressing *pPTPLA*:*PTPLA* were used for comparison in the second and third experiments. n = 3. (C) VLC/LCFA ratio in *pas2-1* and *pas2-1/ptpla* mutants. n = 3. (D) VLCFA levels in three independent *kcr2* mutant liness compared to wild type. n = 3. Significant differences were determined using the student’s t-test: *p<0,05, **p<0,01, ***p < 0.001.

The PTPLA sequence is as divergent from PAS2 sequence as it is from yeast PHS1 (S2 Fig) but it has conserved essential amino acids for the catalytic activity, suggesting a potential function of PTPLA as a 3-hydroxyacyl-CoA dehydratase. The hallmark of the dehydratase deficient mutant is the accumulation of 3-hydroxyacyl-CoA precursors as characterized in the *pas2-1* mutant [5] (Fig 5A). Acyl CoA quantification by LC ESI-MS/MS in root extracts of the *ptpla* mutant confirmed the accumulation of C18, C20 and C22 3-hydroxyacyl-CoAs with respectively 3.0, 4.4, and 1.6 fold the levels observed in wild type (Fig 5A). The 3-hydroxyacyl-CoAs accumulations in *ptpla* were however much lower to that observed in *pas2-1* mutant (Fig 5A, inset). We then investigated if *ptpla* loss-of-function would also reduce VLCFA elongation as observed in *pas2-1*. But contrary to *pas2-1*, the *ptpla* mutant showed a modest but significative increase in VLC/LCFA ratio in seedlings roots but not in the apical part (Fig 5B and S7H Fig). Wild type levels of VLCFA were partially or totally restored in the *ptpla* mutant expressing *pPTPLA*:*PTPLA* construct confirming that the increase in VLCFA content was caused by the loss of PTPLA function. The degree of observable complementation was correlated with the level of *PTPLA* transcripts (S6K Fig). The increase of VLCFA levels induced by the absence of PTPLA function was however dependent on the presence of PAS2 activity since the *ptpla* effect was abolished in *pas2-1/ptpla* double mutant (Fig 5C). These data indicate that PTPLA is first, directly involved in fatty acyl elongation as a 3-hydroxyacyl-CoA dehydratase and second, is involved in regulating PAS2-associated fatty acid elongation.

### VI A specific elongase activity in vascular tissues regulates endodermal VLCFA elongation

The fact that *PTPLA* and *PAS2* had non-overlapping roots expression profiles with respectively vascular tissue/pericycle and endodermis specificity suggests that two different elongase complexes are coexisting in adjacent root cells. To confirm that the vascular/pericycle PTPLA-associated complex was different from the endodermal PAS2-associated complex, we compared the effect of ectopic expression of PTPLA and PAS2 on VLCFA levels. When expressed in the endodermis (under the control of PAS2 promoter), both proteins induced an increase in VLCFA levels (Fig 2C and 2D, S7B Fig) but when expressed in vascular tissue (under the PTPLA promoter) weaker difference with wild type could be observed (S7F Fig). This result indicates that the elongase activity is stronger in the endodermis and that the low vascular/pericycle elongase activity was not caused by a less active PTPLA but rather by limiting quantity of elongase partners, since *PAS2* expression was not sufficient to induce higher VLCFA accumulation.

The presence of a structurally and functionally different PTPLA-associated elongase complex is reinforced by the fact that like PTPLA, KCR2 could not complement the null yeast ortholog mutant *ybr159* [4] and that *pKCR2*:*GUS* was also specifically expressed in vascular tissues in the root (Fig 3R). Interestingly, the *bona* fide KCR1 was expressed in the endodermis like PAS2 (Fig 3M). Fatty acid analysis of *kcr2* mutants showed that VLCFA levels were also increased compared to wild type (Fig 5D and S7E Fig). In conclusion, two elongase complexes with potentially different catalytic properties coexist and more importantly functionally interact in adjacent cells.

## Discussion

In the present study, we identified Arabidopsis PTPLA as a new interacting component of the fatty acyl elongation complex. Several lines of evidence indicate that PTPLA is acting as a 3-hydroxyacyl-CoA dehydratase involved in VLCFA synthesis. First, PTPLA shares significant sequence identity with PAS2 and PHS1, both catalyzing the dehydratase activity required for the VLCFA elongation [5,6]. These three proteins shared the three conserved amino acids determined as essential for the enzymatic activity of the yeast PHS1 protein and constituting the putative active site of the dehydratase [29]. Substitution of Tyr-149 and Glu-156 residues in PHS1 resulted in a loss of growth restoration of *Tet-PHS1*+DOX cells and a complete loss of enzyme activity [29]. Secondly, PTPLA was also interacting in the ER with several elongase subunits. Thirdly, PTPLA was able to restore VLCFAs elongation in the weak *Tet-PHS1*+DOX mutant and its overexpression increased VLCFAs levels in both yeast and plants. Finally, loss of *ptpla* function led to 3-hydroxyacyl-CoA accumulation, which is a hallmark of defective dehydratase activity. However, PTPLA was not able to restore growth of a null *phs1* strain, nor able to complement *pas2* developmental phenotype even when expressed under the control of *PAS2* promoter. PTPLA and PAS2 showed some difference in binding the different KCS enzymes. Ectopic expression of *PTPLA* increased VLCFA levels in wild type background, an effect that is dependent on PAS2 activity. The fact that both proteins were able to interact would suggest that PTPLA might stabilize or activate PAS2 or PHS1 activity. Altogether these results suggest that endogenous PTPLA activity is either very low or present narrower substrate specificity compare to PAS2-dependent elongase complex.

Substrate specificity was previously associated with the first elongation step. In yeast, elegant genetic and biochemical experiments demonstrated the existence of a caliper-like mechanism able to monitor the acyl chain length [6]. In plants, the specificity was associated with specific proteins of the KCS family. For example, *kcs2/daisy-1* and *kcs20* are required for the elongation of C22 VLCFA for cuticular wax and root suberin biosynthesis [32]. KCS18 elongates 20 carbons acyl-chain substrates [3] whereas KCS5 had a preference for 26 carbons acyl-chain substrates [33]. In mammals, similar substrate specificity was also demonstrated for the ELOVL family (ELOVL1–7). For example, ELOVL2 and ELOVL5 catalyze the elongation of polyunsaturated acyl-CoAs with C20–C22 and C18–C20 specificity respectively [34,35]. ELOVL1 and ELOVL4 are responsible for the production of saturated and monounsaturated VLCFAs with lengths respectively of C22-C26 and superior to C28 [35]. No substrate specificity was reported for the other subunits of the elongase complex. The 3-hydroxyacyl-CoA profiles revealed that a larger accumulation of C18-OH in *ptpla* compared to *pas2-1* mutant suggesting that PTPLA would preferentially use C16-CoA substrate. The specificity of the different elongase complexes is most probably determined by specific recruitment of KCS which are known for their different acyl-CoA preferences [30]. The fact that PAS2 and PTPLA did not seem to show the same interactions pattern with the KCS confirmed the existence of different elongase complexes with most probably specific activities.

Besides the substrate specificity, PTPLA differs from PAS2 by its specific expression in the vascular tissues in young seedling roots. Nobusawa et al., showed that PAS2 is present only in the epidermis of leaves and stems [25]. We showed that *PAS2* was also specifically expressed in the endodermis of the primary and secondary roots, as well as at the epidermis of young secondary root tips. *PTPLA* and *PAS2* expressions could match those of several *KCS*. Joubes et al. have shown that KCS family are divided in 8 subclasses with different tissue specificity and at least seven *KCS* genes were found to be strongly expressed in roots [1]. Strikingly, the first reductase genes *KCR1* and its homologue *KCR2* displayed expression patterns in the root reminiscent of those of *PAS2* and *PTPLA* respectively. Besides the similar expression pattern in the root, PTPLA and KCR2 were not able to complement loss of function of their yeast orthologous genes and led when mutated to higher levels of VLCFA in Arabidopsis [4]. A more complete analysis of elongase gene coexpression at different developmental stages or upon different abiotic or biotic stresses could reveal different pattern of expression that would suggest more complex elongase enzyme associations.

**Fig 6:**
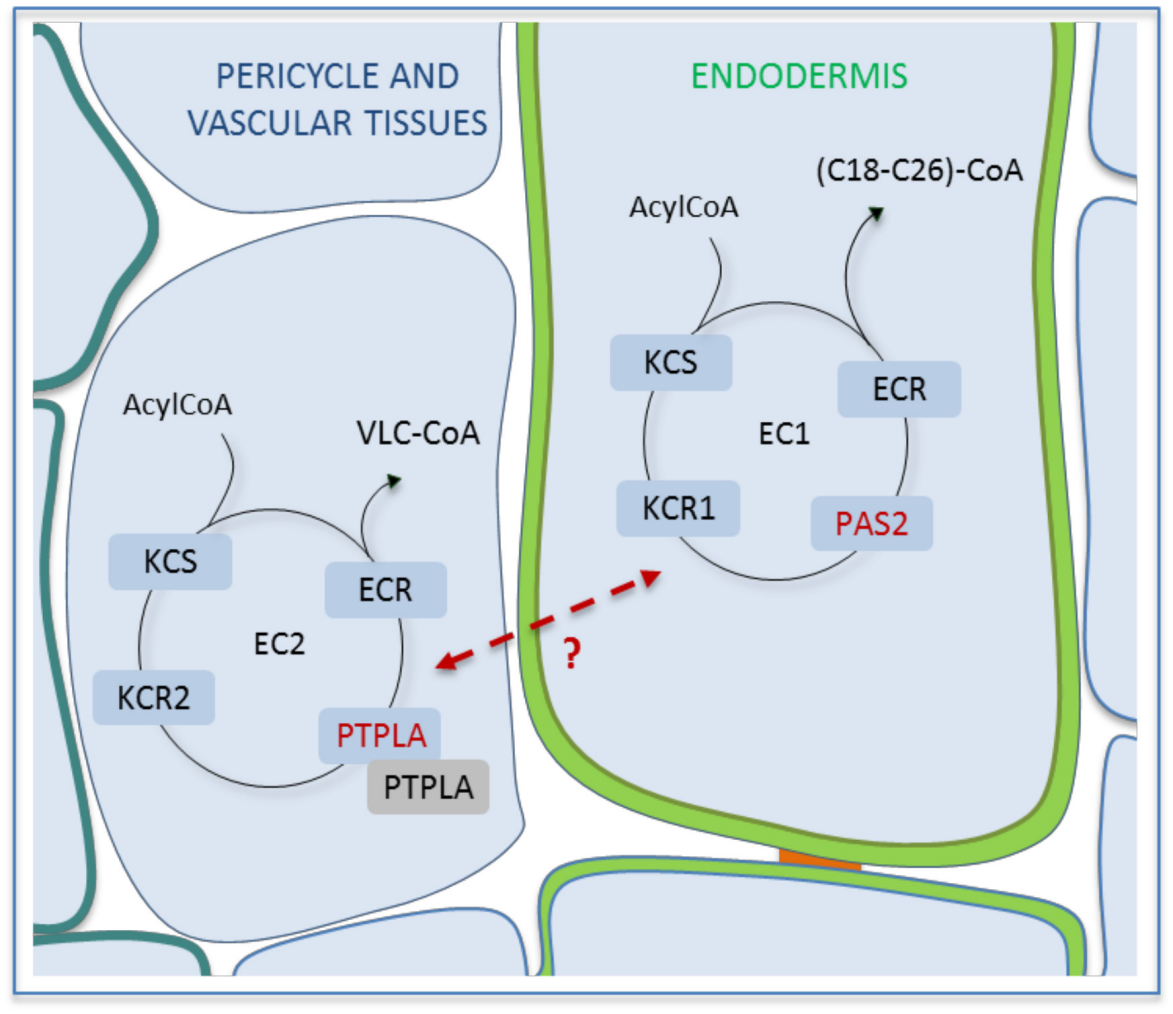
Model of PTPLA mode of action. PTPLA encodes a new dehydratase of the elongase complex specifically localized in the pericycle and vascular tissues that impact the elongase activity in the root endodermis. EC1: PAS2-associated elongase complex, EC2: PTPLA-associated elongase complex. KCS: 3-ketoacyl-CoA synthase. KCR: β-ketoacyl-CoA reductase. ECR: enoyl-CoA reductase. Green thickening: suberin. Blue thickening: vascular tissues. Red band: Caspary band. Red dotted line: non-cell autonomous signal.

We thus propose that PTPLA would be a 3-hydroxyacyl-CoA dehydratase associated with specific elongase complex activity in the vascular tissue and pericycle cells (Fig 6). Contrary to PAS2-based elongase complex in the endodermis that provides most of root VLCFA, PTPLA-based fatty acyl elongation would have a lower activity most likely targeted toward C18-C20 fatty acids. PAS2-based fatty acyl elongation in the endodermis and epidermis would provide VLCFA for the main lipid pools of these tissues, respectively suberin and cuticular waxes [5]. PTPLA-based fatty acyl elongation on contrary would rather promote the synthesis of a regulatory signal modifying PAS2 associated elongase activity. Recent work also identified a regulatory role of epidermal VLCFA by repressing cytokinin synthesis in vascular tissue in a non-cell autonomous [25]. Our present work uncovered a similar regulatory role of VLCFA in the root. Indeed, in absence of PTPLA or KCR2, PAS2-dependent fatty acyl elongation was enhanced suggesting that PTPLA-based elongation in vascular tissue is repressing PAS2-based elongase activity in adjacent endodermal cells.

From these data, we propose a model where vascular tissues express a specific elongase activity (PTPLA-associated) regulating endodermal VLCFA elongation (PAS2-associated) (Fig 6). This regulatory elongase complex would most probably include KCR2 but further more in depth biochemical experiments are necessary to dissect elongase complex composition and stoichiometry. The model suggests the existence of a signal that would be the root counterpart of the model of a non-cell autonomous signal diffusing from the epidermis to the central cylinder of leaves and stems [25]. The nature of the signal is still unknown but cytokinins would be a likely candidate.

## Materials and Methods

### Yeast transformation and growth

The Arabidopsis cDNA library was built in a *Saccharomyces cerevisiae* expression vector pFL61 from young *A*. *thaliana* seedlings (two leaves stage) [36]. The *Tet-PHS1* mutant strain was originating from the R1158 parental strain (*URA*::*CMV-tTA MATa his3-1 leu2-0 met15-0*) carrying the inducible TetO7 system upstream the *PHS1* gene and replacing the endogenous promoter [37] (S1 Fig). *PHS1* was shut off after addition of doxycycline in the medium as described previously [37]. *URA3* gene was disrupted by a nourseothricin resistance cassette in *Tet-phs1* strain *(ura3-*, *nour*^*R*^*)* according to Janke *et al* [38] (primers URA3 used for disruption, LG73 and LG74 see S2 Table) to allow pFL61 selection. After transformation by the lithium acetate procedure [39], yeast were spread out on a SD medium without uracil but with doxycycline (10μg/mL) and 2% of glucose (SD-URA+DOX), then incubated at 28°C during 4–7 days. Among the 698 clones able to grow on the selective medium, PAS2 was expected as a potential suppressor of *Tet-phs1*, PAS2 primers (PAS2F and PAS2R primers S2 Table) were used by PCR to eliminate these clones. Plasmid DNAs from the non-PAS2 remaining yeast colonies were extracted by zymolyase according to the cold spring harbor protocol (Deplancke, 2006) and PCR amplified using primer LG69 and LG70 (S2 Table). All these PCR products were sequenced by GenoScreen and all the sequences were blasted into the TAIR database. PHS1 was cloned in pFL61 to use as positive control (primers CM22 and CM23). For kinetic growth, 0,3U.OD_600nm_ of the different strains (mix of 4 clones per strain) from a saturated preculture were inoculated in 50mL and 150mL of liquid SD-URA+DOX medium respectively and agitated at 28°C for 4 days. OD_600nm_ was monitored until the culture reached stationary phase.

### Plant material and construction of plant expression vector

A T-DNA insertion mutant line for *PTPLA* (At5g59770) was identified using the Arabidopsis Gene Mapping Tool (http://signal.salk.edu/cgi-bin/tdnaexpress), and the seed stock (SALK_077395) was obtained from the NASC. Homozygous lines were selected after genomic DNA extraction and by PCR screening for the presence of a T-DNA insertion (CM14 and LB1.3 primers) and the absence of the *PTPLA* intact gene (LG79 and LG103 primers) (S2 Table). The *pas2-1* mutant is an ethyl methane sulfonate allele in Col0 background that was maintained as heterozygous stocks. For all RT and lipid analysis, the homozygous *pas2-1 mutants* were selected based on the characteristic pepper-like shape of mutant seeds. The *pas2-1/ptpla* double mutant was generated by crossing the heterozygous *pas2-1* mutant with homozygous *ptpla* (SALK_077395). The F1 was genotyped by *bstnI* digestion of a specific *PAS2* PCR product (PAS2-1F and PAS2-1R specific primers) to detect *pas2-1* mutation and with the previous CM14/LB1.3 primers to detect *ptpla* T-DNA insertion. *PAS2* and *PTPLA* promoters used for the following constructs correspond to 2000 bp and 1250pb genomic sequence upstream the ATG codon of *PAS2* and *PTPLA* genes respectively. To generate the *pPAS2*:*GFP-PTPLA*, *pPAS2*:*GFP-PAS2*, *pPAS2*:*PTPLA*, *pPAS2*:*PAS2*, *pPTPLA*:*PAS2*, pPTPLA:PTPLA constructs, PAS2 (-2000 bp from the ATG) and PTPLA promoters (-1250 bp from the ATG) were first cloned into pB7WGF2 (*pPAS2*:*GFP-GTW*; *pPTPLA*:*GFP-GTW*) or pB2GW7 (*pPTPLA*:*GTW*) vectors by restriction enzymes to replace the 35S promoter. PCR amplifications of the promoters with primers containing restriction enzyme sites of HindIII (LG116) and SpeI (LG117) were used for pPAS2 cloning and restriction sites of SacI (LG123) and SpeI (LG124) were used for pPTPLA cloning. In parallel, the coding DNA sequence (CDS) of *PTPLA* was amplified with (primers CM02/CM03) or without (primers CM02/CM04) the stop-codon using the full-length cDNA (G61261: cDNA into pENTER223) and were transferred into pDONR207 by BP cloning and then recombined by LR into the previous vectors according to the Invitrogen^™^ protocol. The same procedure was done with the *PAS2* CDS, the stop and non-stop versions already cloned in pDONR207 [5]. PTPLA G61261 was also recombined into 35S::gtw pGWB2 [40], 35S::GFP-gtw pGWB6 [40] and *35S*::*mCherry-gtw* [41] to generate 35S:PTPLA, 35S:GFP-PTPLA and 35S:mCherry-PTPLA respectively. Finally, the *pPAS2*:*GFP-PTPLA*, *pPAS2*:*GFP-PAS2*, *pPAS2*:*PTPLA*, *pPAS2*:*PAS2*, *pPTPLA*:*PAS2*, *pPTPLA*:*PTPLA*, *35S*:*PTPLA* and *35S*:*GFP-PTPLA* generated constructs were transformed into heterozygous *pas2-1/+* by the floral-dip method (Clough and Bent, 1998). All the experiments described in this work were carried out on homozygous T3 plants.

Concerning the *pPTPLA*:*mRFP1* and *pPTPLA*:*GUS* constructs, 1250bp PTPLA promoter was inserted into *pDONR207* to generate *pDONR207*:*pPTPLA* (LG94 and LG96 BP primers) and recombined into pGWB553 and pGWB3 upstream of the mRFP or GUS markers respectively by LR cloning. The *pPAS2*:*GFP* construct was obtained by LR recombination of a GFP-stop cDNA (pDONR207:GFP) into pB7FWG2 vector (*pPAS2*:*GTW-GFP*). Both *pPTPLA*:*mRFP1* and *pPAS2*:*GFP* constructs were transformed together into Col0 to observe the localization of *PTPLA* and *PAS2* expression. Finally, the *pPAS2*:*GUS* constructs was obtained by recombination of 1500bp PAS2 promoter into the pMDC162 vector. The *pKCR1*:*GUS* construct was obtained from Jerome Joubès, *pKCR2*:*GUS* construct from Ljerka Kunst and the *kcr2* mutant by Frédéric Beaudoin.

### Lipid Analysis

Plants were vertically grown on MS Arabidopsis medium for 14 days [42]. Roots were quickly sampled by cutting below the hypocotyl. Overexpressor and complemented yeast strains from a saturated preculture were inoculated in 50mL (LB-URA) or 150mL (LB-URA+DOX) respectively and 150 OD units were sampled at about 26h and 47h after inoculation respectively so that cells would be in exponential phase of the growth culture with an OD600 between 4 and 6. The cultures were centrifuged 10 minutes at 3000rpm and cells were washed with cold sterilized water. Both yeasts and roots were immediately frozen after sampling at -80°C for more than 1 hour and then lyophilized. For quantification of the fatty acids methyl ester (FAMES), around 2 mg of each dry sample were used according to the Li et al protocol by GC-MS [43]. Three technical and two or three biological replicates per sampled were analyzed. Quantification of acylCoA of Arabidopsis and yeast material by LC ESI-MS/MS required 100mg of frozen fresh roots and 1 OD unit of frozen yeast. Roots and yeasts were grown as described before for the FAMES analysis. AcylCoA extraction and LC separation was carried out as reported by [44]. The MS multi-reaction monitoring was done as described by Haynes *et al*. [45]. Five replicates per samples were analyzed.

### Transient infiltration of *N*. *benthamiana* leaves

For co-expression experiments and BiFC interactions, the vectors were transformed into *Agrobacterium tumefaciens* and inoculated in *Nicotiana benthamiana* leaves of one month-old plants. Agrobacterium carrying clones of interest were grown overnight at 28°C in 5 ml LB medium with appropriate antibiotics. Aliquots from the overnight cultures were resuspended in 10 mM MgCl_2_ and 1 mM 2-(N-morpholine)-ethanesulphonic acid (MES) to obtain a final OD_600nm_ of 0,5 for tobacco leaf infiltration. The BiFC experiments were done twice. Split luciferase was carried out according to van Leene [46].

### Cytologic and microscopic analysis

For analysis of the GUS (β-glucuronidase) activity, Arabidopsis seedling of 14 days were incubated at 37°C with 1mg/mL X-Gluc (5-bromo-4-chloro-3-indolyl-D-glucuronic acid) and in a GUS reaction buffer (100 mM sodium phosphate buffer, pH7.2, 10mM sodium EDTA, 0.1% Triton X-100, 1mM potassium ferricyanide, 1mM potassium ferrocyanide), after 2x5 minutes of vacuum infiltration. The stained seedling were cleared by successive ethanol washes from 30% until 70% and visualized under a Zeiss light macroscope (Axiozoom). Observations of XFP markers were carried out using a Leica SP5 AOBS confocal laser microscope using either a PL APO 20x0.70 NA or 63x1.20 NA water-immersion objectives. GFP and mCherry/mRFP1 fluorescence were respectively recorded after an excitation at 488 and 561 nm (Argon laser and laser diode respectively) and a selective emission band of 495–550 nm and 600–625 nm. YFP fluorescence was recorded after an excitation at 514 nm (Argon laser) and a selective emission band of 520–565 nm. Autofluorescence of the chloroplast was excited by the Argon laser (488 or 514 nm) and recorded with a selective emission band of 650–700 nm. Root length were measured with the segmented line tools of ImageJ software and the number of lateral root was counted under a binocular.

### Real-time RT-qPCR conditions and analysis

The total RNAs were extracted from 14 day-old *Arabidopsis thaliana* roots using the RNeasy^®^ Plant Mini Kit (Qiagen) according to the manufacturer’s instructions. The Reverse Transcription was performed with reverse transcriptase (Fermentas) each reaction containing 1ug of total RNA. Quantitative PCR gene-specific primers were designed to span the introns. The qPCR primer sequences specific to PTPLA (CM35 and CM36), PAS2 (PAS2 Q-PCR1 F and PAS2 Q-PCR1 R) and AT2G28390 reference primers [47] are resumed in S2 Table. Quantitative PCR was performed on a CFX96 machine from Biorad under the following conditions: 3min at 95°C follow by 35 cycles of 10s at 95°C, 20s at 60°C and 20s at 72°C, and finally 95°C for 30s. The data were analyzed with the CFX96 manager Biorad 3.0 software.

## Supporting Information

S1 Fig (A) The yeast Tet-regulated promoter system. The *Tet-PHS1* strain was obtained by replacing in R1158 strain the *PHS1* promoter with the KanR-tetO7-TATA cassette and the tTA* transactivator was integrated at the *URA3* locus. In absence of doxycycline, tTA* protein binds TetO7 promoter and lead to *PHS1* transcription. Addition of doxycycline prevents tTA* binding to TetO7 promoter and, thus, prevents *PHS1* transcription. *NGC1*, next gene on chromosome. (Modified from [37]). (B) A. thaliana PTPLA does not complement the yeast phs1D mutant. *PHS1* and *PAS2* but not PTPLA rescue the lethality of the *phs1*::*KanMX4* knock out strain. *Mat a* and *Mat a phs1*::*KanMX4* clones complemented by the yeast *PHS1* gene cloned into a pADH-URA vector [5] were transformed with yeast *PHS1*, Arabidopsis *PAS2*, Arabidopsis PTPLA or an empty pADH-LEU vector. Transformed clones were streaked on synthetic complete medium (SC, ForMedium Ltd, UK) in the presence or in the absence of 2 g/l 5-fluoroorotic-acid (FOA) as described in Bach et al. 2008 [5].
(PDF)

S2 Fig Sequence alignement of the PTPLA, PAS2 and PHS1. The three proteins share conserved amino acids in yellow essential for the dehydratase activity of PHS1 [29], the transmembrane segments (grey boxes) and the ER retention signal, KXKXX and KKXX (blue boxes). PTPLA is closely related to PAS2 and PHS1 with respectively 32% and 35% of identity.
(PDF)

S3 Fig PHS quantification in yeast modified for PHS1 expression. R1158 wild-type strain and *Tet-PHS1* mutant strain transformed with the pFL61 empty vector, PTPLA or PAS2 were grown at 28°C over night in YPD medium. Precultures were transfered into YPD in the presence or absence of 10μg/ml of DOX to reach 0,3 unit of D0_600_ and agitated at 28°C during six hours. Aliquots of cell suspensions were samplied and lyophilised to obtain 100mg of dry yeast extract. LCB species were extracted, treated with o-phthalaldehyde, and separated by reverse-phase HPLC as described in [48]. PHS_20_ was quantified using D_20_ (D-erythro-sphinganine) as an internal control.
(PDF)

S4 Fig FAMES analysis in yeast expressing PTPLA, PAS2 and PHS1. (A) FA profile in *Tet-PHS1* mutant expressing PTPLA, PAS2 and PHS1 (*Tet-PHS1*/) compared to R1158. n = 5–12. (B) FA profile in R1158 ectopically expressing or not PTPLA, PAS2 and PHS1. n = 9–15.
(PDF)

S5 Fig 3-hydroxyacyl-CoA quantification in yeast modified for PHS1 expression. Wild type strain R1158, *Tet-PHS1* strain expressing empty vector (*Tet-PHS1*), or expressing PAS2 or PTPLA were cultivated in presence of DOX.
(PDF)

S6 Fig Expression analysis of *PTPLA*. (A-C) Ectopic expression of GFP-PTPLA under the control of PAS2 (A, B) or *35S* promoters (C). (D-F) Expression of *GFP-PAS2* under the control of *pPAS2* 2kb promoter in root (D) and leaves (E). Expression of *GFP-PAS2* under the control of 1kb *pPAS2* promoter (Bach, 2008) (F). Scale: 50μm. (G-J) Quantitative RT-PCR of *PTPLA* mRNA (G-H) *and PAS2* mRNA (I-J) in *ptpla*, *pas2-1* and *pas2-1/ptpla* double mutant and in three independent transgenic lines expressing *PTPLA* under *pPAS2* promoter in Col0 and *pas2-1* background. (K) Quantitative RT-PCR of *PTPLA* mRNA and *PAS2* mRNA in three independent *ptpla* mutant lines expressing *pPTPLA*:*PTPLA* construct. Significant differences with Student’s t-test are indicated: ***p ≤ 0.001.
(PDF)

S7 Fig Total FA profile from Arabidopsis thaliana seedlings. Total FAMES analysis of (A) roots from *pas2-1* and *pas2-1/ptpla* double mutants and in *pas2-1* expressing *pPAS2*:*PTPLA* construct; (B) roots from *ptpla*, *ptpla* complemented by *pPTPLA;PTPLA 1* and wild type (Col0) lines expressing *pPAS2*:*PTPLA* construct; (C) roots from ptpla and three independent *pPTPLA*:*PTPLA* complementation lines; (D) roots from pas2-1 and two independent pPAS2:PAS2 complementing lines; (E) roots from three independent *kcr2* mutant lines; (F) roots from wild type (Col0) expressing *pPTPLA*:*PTPLA* and *pPTPLA*:*PAS2* constructs; (G) roots from *35S*:*PTPLA*, *pPAS2*:*PTPLA* and *pPAS2*:*PAS2* independent transgenic lines and (H) seedling apical part of *pas2*, *ptpla* and double *pas2/ptpla* mutants. The analysis were done on roots or apical part of 14 days-old seedlings. n = 3. Insets show C20-C26 contents.
(PDF)

S8 Fig Characterization of *ptpla* mutant. (A) *PTPLA* gene structure is represented with ATG codon (0), exons (dark filled boxes), introns (grey lines), untranslated region (UTR, grey filled boxes) and T-DNA insertion (salk077395). Scale: 100bp. Blue arrows represent PCR primers; (B) PCR analysis on genomic DNA of different *ptpla* mutant segregants with insertion specific primers (upper panel), primers on both side of the insertion (middle panel) and EF1a control primers (lower panel); (C) qRT-PCR of *PTPLA* transcript in *ptpla* mutant compared to wild type with primers upstream and downstream from the T-DNA insertion.
(PDF)

S9 Fig Effect of *ptpla* mutation on root development. (A) Primary root length of 14 day-old seedlings and (B) the corresponding lateral root density. n = 35–37. Significant differences were determined using the student’s t-test: *p<0,05, **p<0,01, ***p < 0.001.
(PDF)

S10 Fig BiFC interaction between PTPLA, PAS2 and the KCS of the elongase complex. BiFC interactions in *N*.*benthamiana* epidermal cells of *35S*:*NYFP-PTPLA* or *35S*:*NYFP-PAS2* and *35S*:*CYFP-KCSX* (with X = 1 to 20). Chloroplast autofluorescence is shown in blue. Scale: 50μm.
(PDF)

S11 Fig PTPLA interaction assays by split-luciferase. PTPLA interaction assay was carried out between either two PTPLA or between PTPLA and PAS2, CER10 or a negative control (DPL1). The red line indicates the threshold of interaction corresponding to the constitutive fluorescence observed for all positive and negative tested samples. The red line indicates arbitrary negative threshold value for interaction.
(PDF)

S1 Table Segregation analysis of transgenic lines expressing *PTPLA* or *PAS2*. Heterozygotes *pas2-1/+* mutants transformed with different constructs with *PTPLA* or *PAS2* coding sequence under the control of *PAS2* or 35S promoters. Segregation was analyzed in T2 progeny of several independent transgenic lines (column 2) and only those segregating *pas2-1* mutants were kept for analysis. Complementation was scored positive (column 3) when 25% segregating seedlings showed *pas2-1* phenotype (column 4). The constructs expressing *PAS2* with or without the *GFP* under its own promoter were used as control lines. Homozygote *pas2-1/pas2-1* complemented lines showed wild-type phenotype (data not shown) and were identified by the *bstnI* CAPS marker (G622A substitution in the coding sequence).
(PDF)

S2 Table Primers used for PCR cloning and quantitative PCR analysis. The sequences in bold are not specific to the target gene.
(PDF)

## Abbreviations

DOX: doxycycline
ECR: enoyl-CoA reductase
ER: endoplasmic reticulum
FA: fatty acid
FAE: *FIDDLEHEAD* gene
FAMES: fatty acid methyl ester
GUS: β-glucuronidase
HCD: 3-hydroxyacyl-CoA dehydratase
HPLC: high-performance liquid chromatography
KCR: β-Ketoacyl-CoA reductase
KCS: 3-keto-acyl-CoA synthase
LCB: long chain base
NOUR: nourseothricin
PAS: *PASTICCINO* genes
PCR: polymerase chain reaction
PGK: phosphoglycerate kinase promotor
PHS: phytosphingosine
PHS1: yeast 3-hydroxyacyl-CoA dehydratase
PTPLA: protein tyrosine phosphatase like A
Tet: titrable promoter TetO7
VLCFA: very-long-chain fatty acid
