# Supplementary figures and images for: Dual Fatty Acid Elongase Complex Interactions in Arabidopsis
Source: PLoS One. 2016 Sep 1;11(9):e0160631. doi: 10.1371/journal.pone.0160631 (PMC5008698; doi:10.1371/journal.pone.0160631)

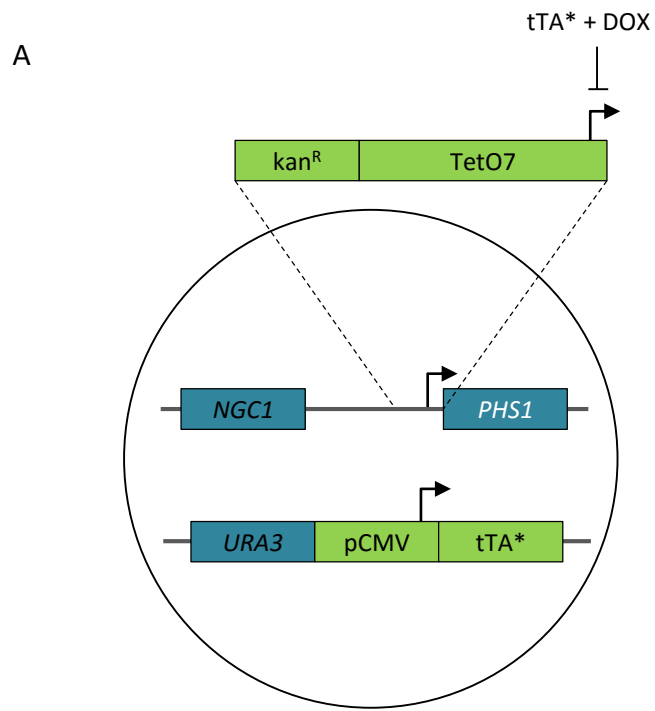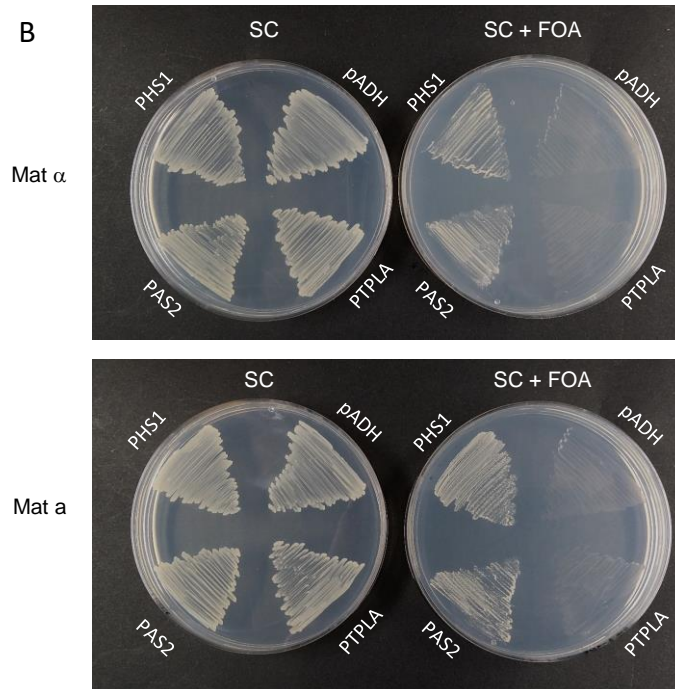

S1 Fig

Supplement: S1 Fig — (A) The yeast Tet-regulated promoter system. The Tet-PHS1 strain was obtained by replacing in R1158 strain the PHS1 promoter with the KanR-tetO7-TATA cassette and the tTA* transactivator was integrated at the URA3 locus. In absence of doxycycline, tTA* protein binds TetO7 promoter and lead to PHS1 transcription. Addition of doxycycline prevents tTA* binding to TetO7 promoter and, thus, prevents PHS1 transcription. NGC1, next gene on chromosome. (Modified from [37]). (B) A. thaliana PTPLA does not complement the yeast phs1D mutant. PHS1 and PAS2 but not PTPLA rescue the lethality of the phs1::KanMX4 knock out strain. Mat a and Mat a phs1::KanMX4 clones complemented by the yeast PHS1 gene cloned into a pADH-URA vector [5] were transformed with yeast PHS1, Arabidopsis PAS2, Arabidopsis PTPLA or an empty pADH-LEU vector. Transformed clones were streaked on synthetic complete medium (SC, ForMedium Ltd, UK) in the presence or in the absence of 2 g/l 5-fluoroorotic-acid (FOA) as described in Bach et al. 2008 [5]. (PDF) [file pone.0160631.s001.pdf]

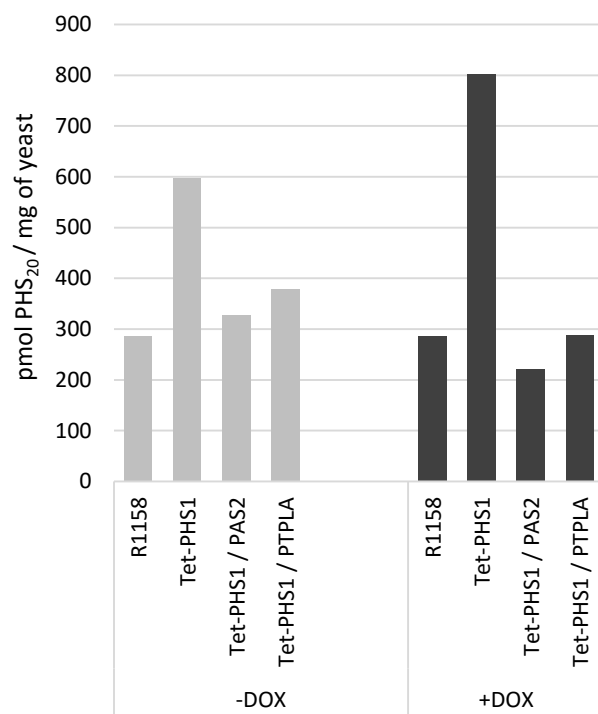

S3 Fig

Supplement: S3 Fig — R1158 wild-type strain and Tet-PHS1 mutant strain transformed with the pFL61 empty vector, PTPLA or PAS2 were grown at 28°C over night in YPD medium. Precultures were transfered into YPD in the presence or absence of 10μg/ml of DOX to reach 0,3 unit of D0600 and agitated at 28°C during six hours. Aliquots of cell suspensions were samplied and lyophilised to obtain 100mg of dry yeast extract. LCB species were extracted, treated with o-phthalaldehyde, and separated by reverse-phase HPLC as described in [48]. PHS20 was quantified using D20 (D-erythro-sphinganine) as an internal control. (PDF) [file pone.0160631.s003.pdf]

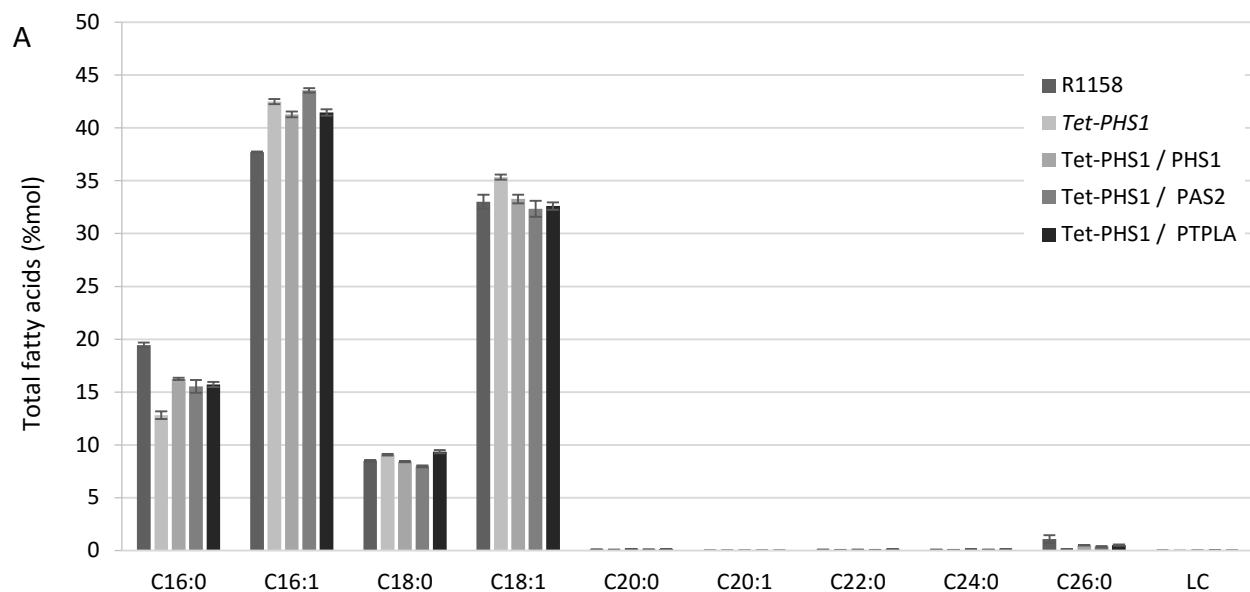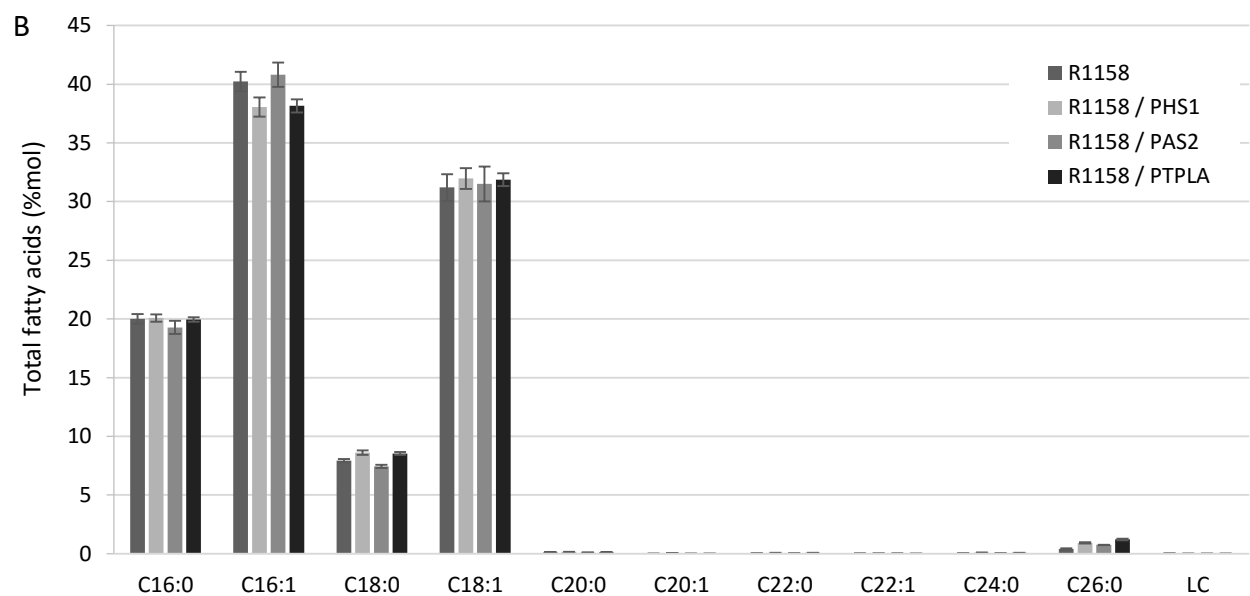

S4 Fig

Supplement: S4 Fig — (A) FA profile in Tet-PHS1 mutant expressing PTPLA, PAS2 and PHS1 (Tet-PHS1/) compared to R1158. n = 5–12. (B) FA profile in R1158 ectopically expressing or not PTPLA, PAS2 and PHS1. n = 9–15. (PDF) [file pone.0160631.s004.pdf]

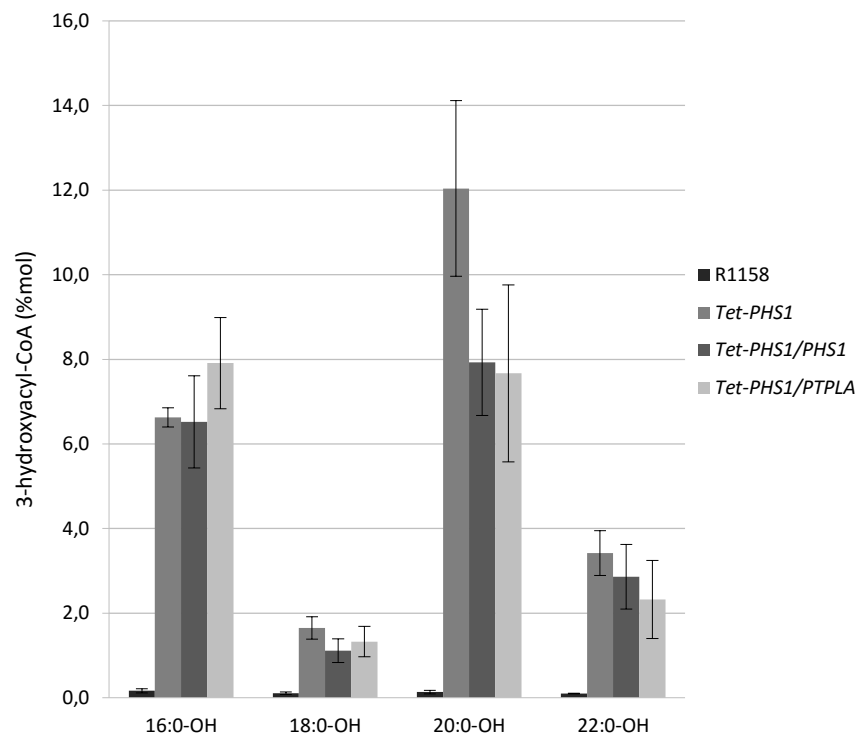

S5 Fig

Supplement: S5 Fig — Wild type strain R1158, Tet-PHS1 strain expressing empty vector (Tet-PHS1), or expressing PAS2 or PTPLA were cultivated in presence of DOX. (PDF) [file pone.0160631.s005.pdf]

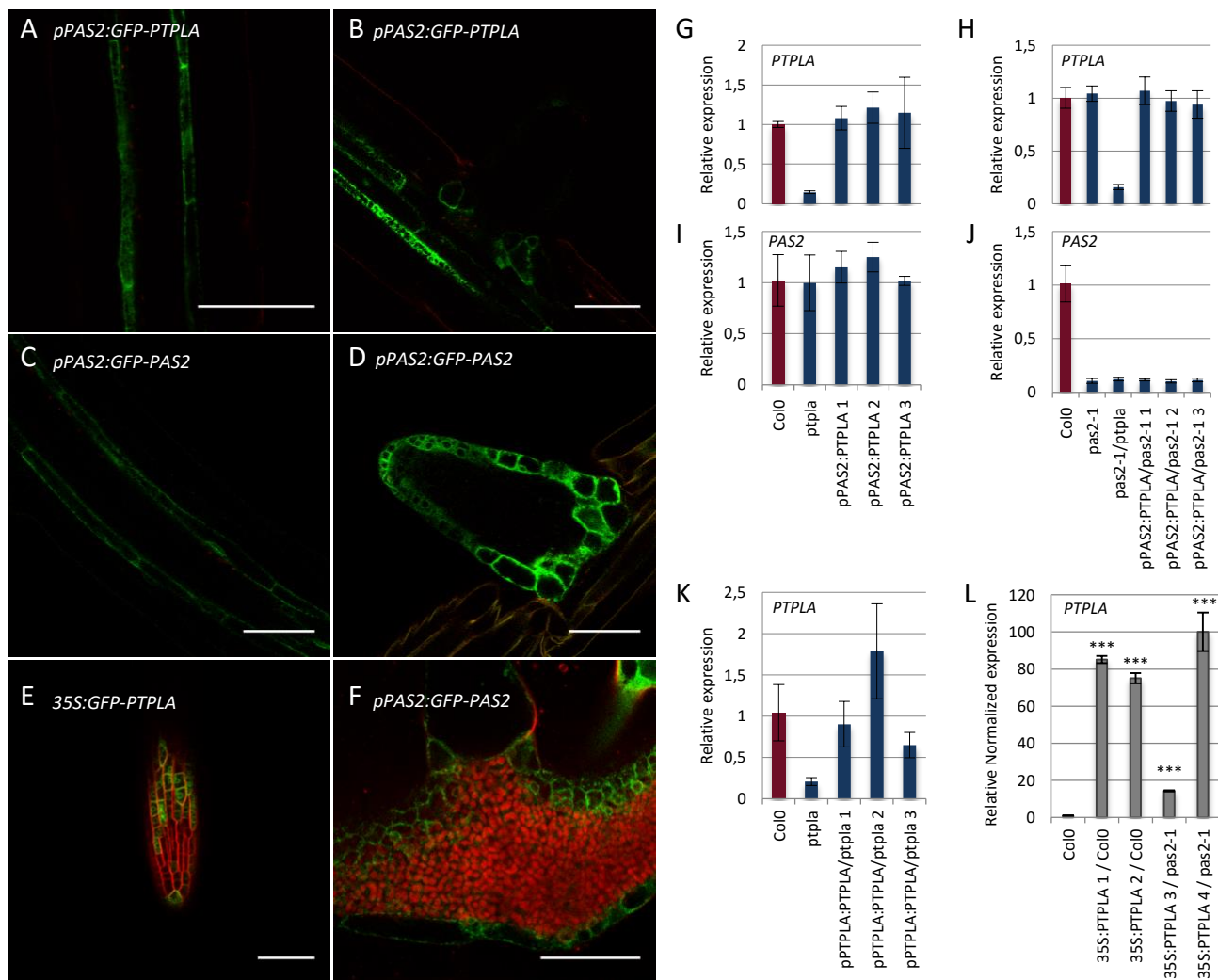

S6 Fig

Supplement: S6 Fig — (A-C) Ectopic expression of GFP-PTPLA under the control of PAS2 (A, B) or 35S promoters (C). (D-F) Expression of GFP-PAS2 under the control of pPAS2 2kb promoter in root (D) and leaves (E). Expression of GFP-PAS2 under the control of 1kb pPAS2 promoter (Bach, 2008) (F). Scale: 50μm. (G-J) Quantitative RT-PCR of PTPLA mRNA (G-H) and PAS2 mRNA (I-J) in ptpla, pas2-1 and pas2-1/ptpla double mutant and in three independent transgenic lines expressing PTPLA under pPAS2 promoter in Col0 and pas2-1 background. (K) Quantitative RT-PCR of PTPLA mRNA and PAS2 mRNA in three independent ptpla mutant lines expressing pPTPLA:PTPLA construct. Significant differences with Student’s t-test are indicated: ***p ≤ 0.001. (PDF) [file pone.0160631.s006.pdf]

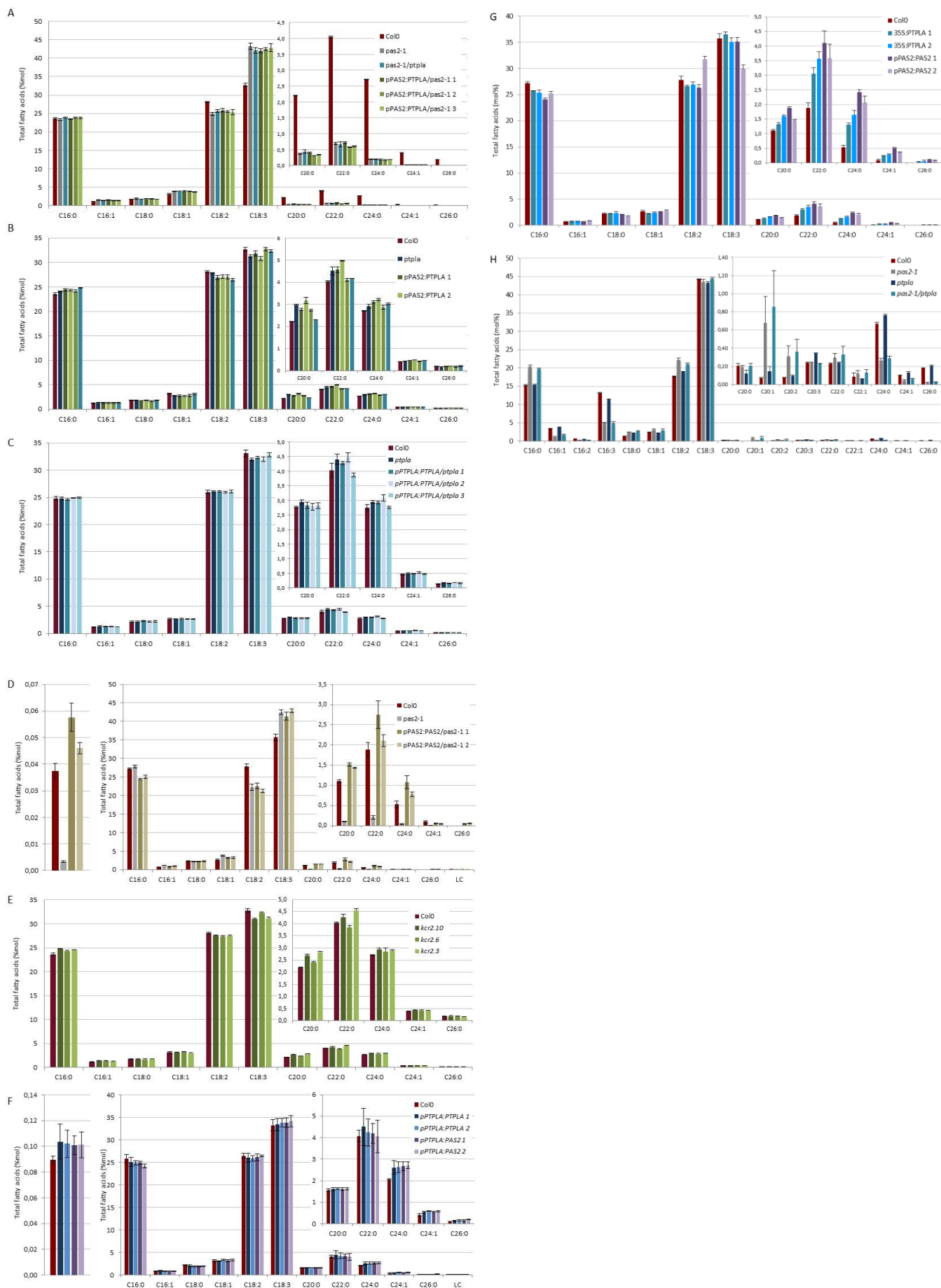

S7 Fig

Supplement: S7 Fig — Total FAMES analysis of (A) roots from pas2-1 and pas2-1/ptpla double mutants and in pas2-1 expressing pPAS2:PTPLA construct; (B) roots from ptpla, ptpla complemented by pPTPLA;PTPLA 1 and wild type (Col0) lines expressing pPAS2:PTPLA construct; (C) roots from ptpla and three independent pPTPLA:PTPLA complementation lines; (D) roots from pas2-1 and two independent pPAS2:PAS2 complementing lines; (E) roots from three independent kcr2 mutant lines; (F) roots from wild type (Col0) expressing pPTPLA:PTPLA and pPTPLA:PAS2 constructs; (G) roots from 35S:PTPLA, pPAS2:PTPLA and pPAS2:PAS2 independent transgenic lines and (H) seedling apical part of pas2, ptpla and double pas2/ptpla mutants. The analysis were done on roots or apical part of 14 days-old seedlings. n = 3. Insets show C20-C26 contents. (PDF) [file pone.0160631.s007.pdf]

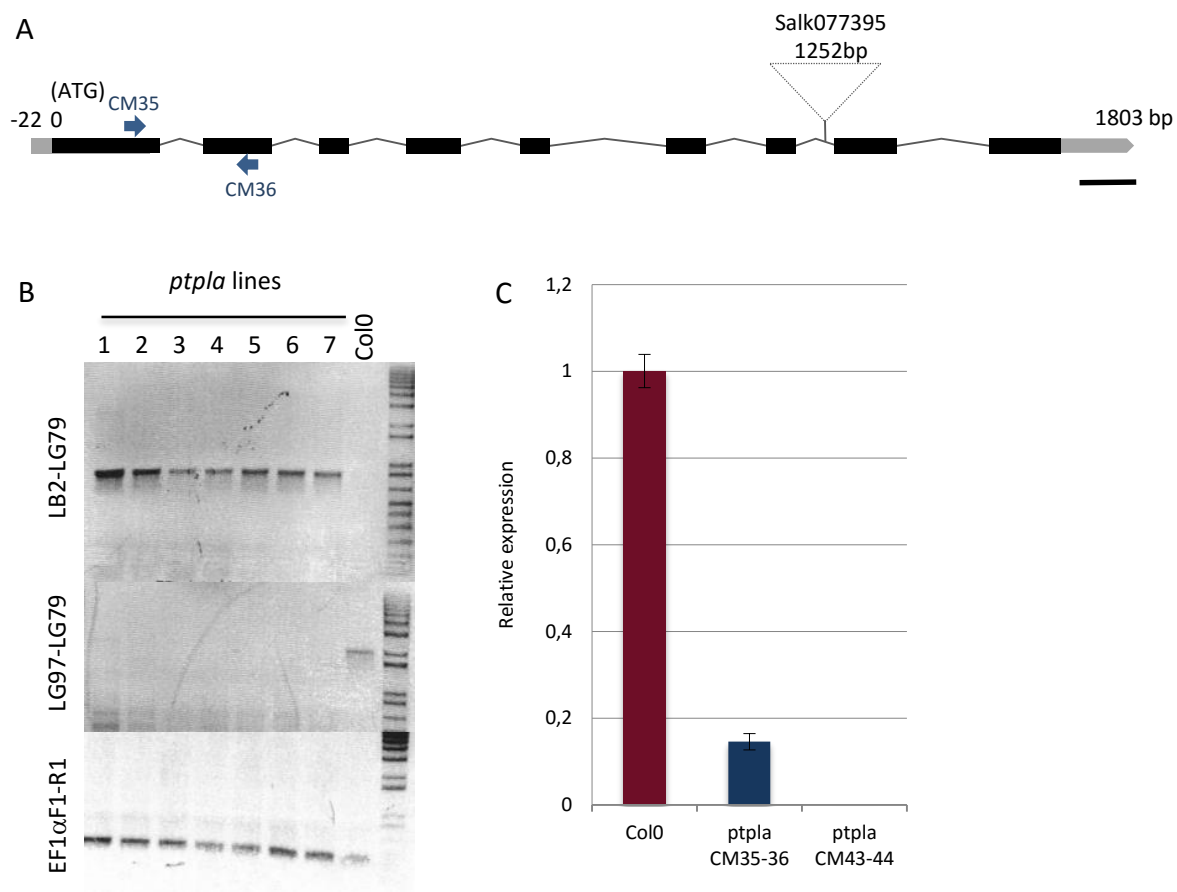

S8 Fig

Supplement: S8 Fig — (A) PTPLA gene structure is represented with ATG codon (0), exons (dark filled boxes), introns (grey lines), untranslated region (UTR, grey filled boxes) and T-DNA insertion (salk077395). Scale: 100bp. Blue arrows represent PCR primers; (B) PCR analysis on genomic DNA of different ptpla mutant segregants with insertion specific primers (upper panel), primers on both side of the insertion (middle panel) and EF1a control primers (lower panel); (C) qRT-PCR of PTPLA transcript in ptpla mutant compared to wild type with primers upstream and downstream from the T-DNA insertion. (PDF) [file pone.0160631.s008.pdf]

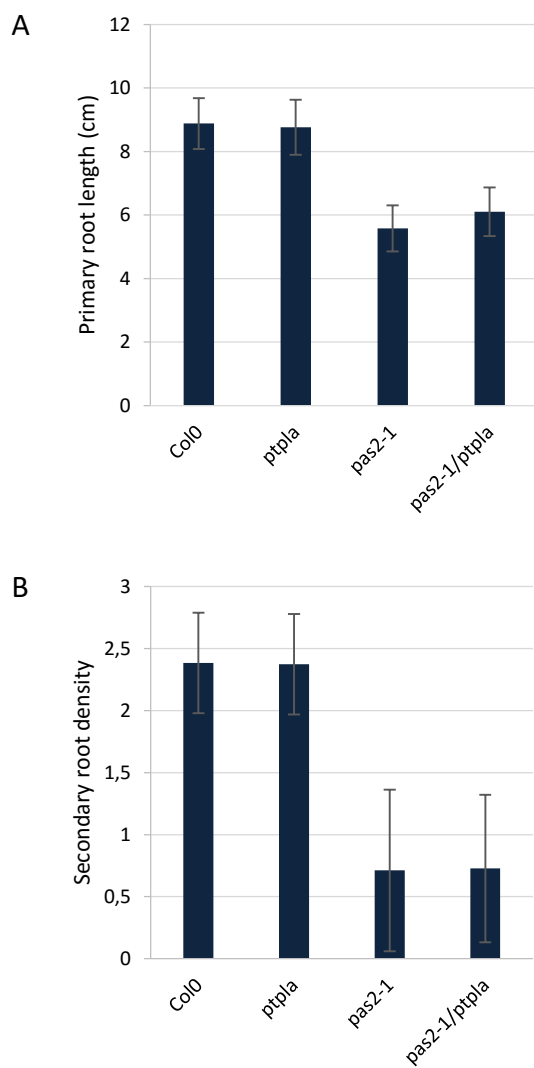

S9 Fig

Supplement: S9 Fig — (A) Primary root length of 14 day-old seedlings and (B) the corresponding lateral root density. n = 35–37. Significant differences were determined using the student’s t-test: *p<0,05, **p<0,01, ***p < 0.001. (PDF) [file pone.0160631.s009.pdf]

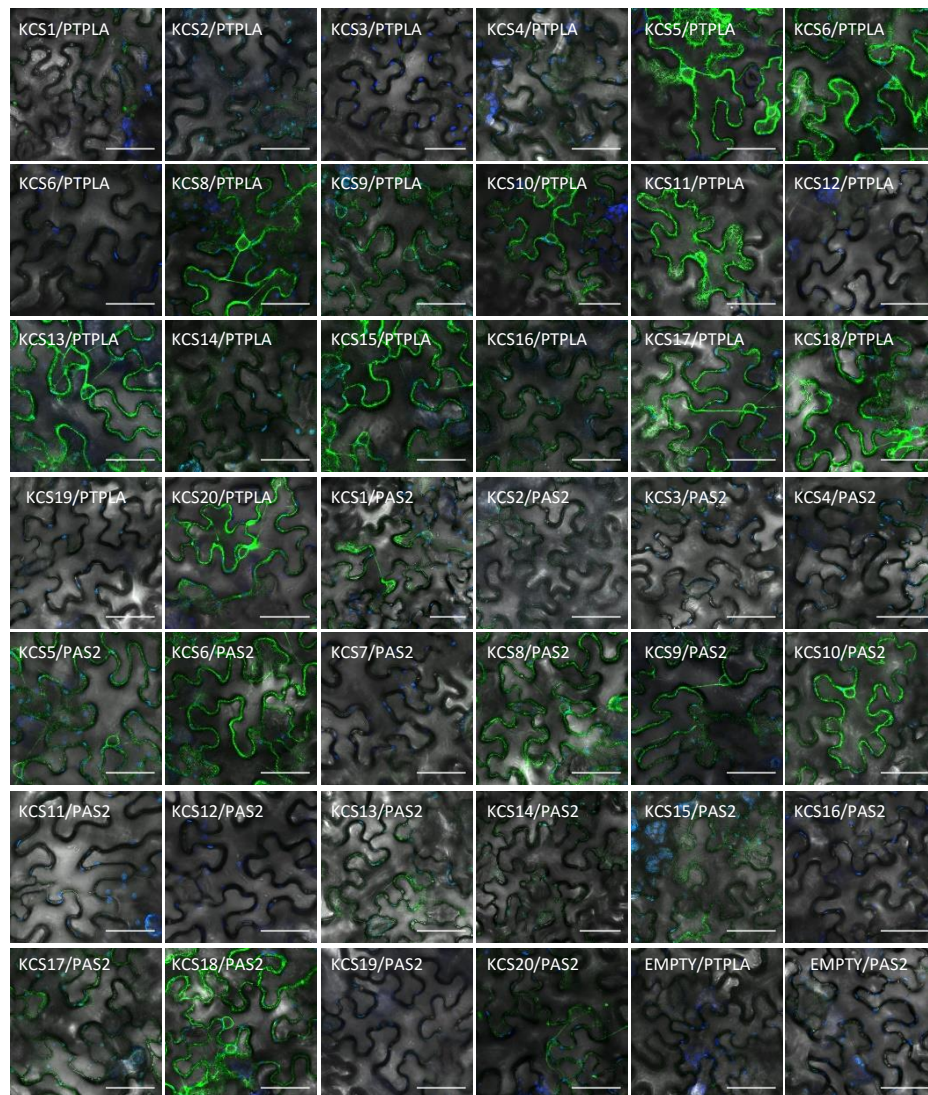

S10 Fig

Supplement: S10 Fig — BiFC interactions in N.benthamiana epidermal cells of 35S:NYFP-PTPLA or 35S:NYFP-PAS2 and 35S:CYFP-KCSX (with X = 1 to 20). Chloroplast autofluorescence is shown in blue. Scale: 50μm. (PDF) [file pone.0160631.s010.pdf]

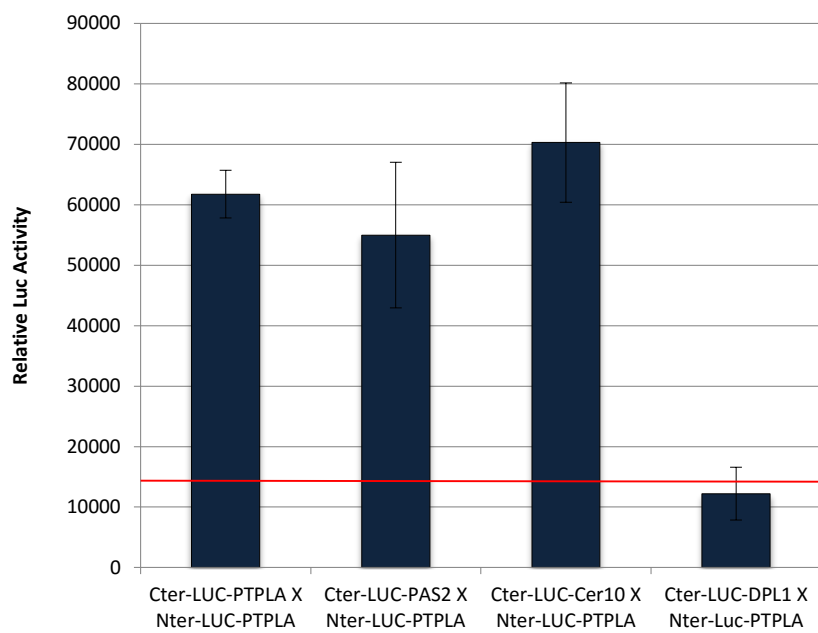

S11 Fig

Supplement: S11 Fig — PTPLA interaction assay was carried out between either two PTPLA or between PTPLA and PAS2, CER10 or a negative control (DPL1). The red line indicates the threshold of interaction corresponding to the constitutive fluorescence observed for all positive and negative tested samples. The red line indicates arbitrary negative threshold value for interaction. (PDF) [file pone.0160631.s011.pdf]
