# Supplementary material for: Dual Fatty Acid Elongase Complex Interactions in Arabidopsis
Source: PLoS One. 2016 Sep 1;11(9):e0160631. doi: 10.1371/journal.pone.0160631 (PMC5008698; doi:10.1371/journal.pone.0160631)
Supplement: S2 Fig — The three proteins share conserved amino acids in yellow essential for the dehydratase activity of PHS1 [29], the transmembrane segments (grey boxes) and the ER retention signal, KXKXX and KKXX (blue boxes). PTPLA is closely related to PAS2 and PHS1 with respectively 32% and 35% of identity. (PDF) [file pone.0160631.s002.pdf]

```
        1                                                     50
PTPLA   ----MSPFVK FYLFSYNFLQ ASAWAISLLI ILNSFLSNKT IISAYASAGF
 PAS2   MAGFLSVVRR VYLTLYNWIV FAGWAQVLYL AITT-LKETG YENVYDAIEK
 PHS1   MSKKLASPLS -FLPLYNLLS AVGWSYLLYL VI-SLYPKVG QPAFFYQTKN

        51                                                   100
PTPLA   LISLFQTAAV LEVLHGAIGI VPSGFLSPLM QWSGRTHFIL AIVGQIKEVQ
 PAS2   PLQLAQTAAV LEILHGLVGL VRSPVSATLP QIGSRLFLTW GILYSFPEVR
 PHS1   VATLVQCGAI IEIINSFLGV VRSPLLTTVA QVSSRLLVVL GIFQLLPNTS

        101                                                  150
 PTPLA  --DSPWLSIT LVAWCIGEMI RYPHYAFT-C L-GRCPYWLT YLRYTGFIVI
 PAS2   --SHFLVTSL VISWSITEII RYSFFGFKEA L-GFAPSWHL WLRYSSFLLL
 PHS1   GVQSVVYISL LLAWSITEIV RYLYYFFMLV FKNGAPKILI LLRYNLFWIL

        151                                                  200
PTPLA   YPTGLVGELL IMYKALPYVK ERNLYANFFS VFPFSYYDFL WAVLLVYPFL
 PAS2   YPTGITSEVG LIYLALPHIK TSEMYSVRMP NILNFSFDFF YATILVLAIY
 PHS1   YPTGVASELR IIYCALNAAE SQ--YSLLYK RIL------- ---IAAMLAY

        201                              232
PTPLA   WL---KLYLQ LFKQRKSKLG KSKKLHGKRK RM
 PAS2   VPGSPHMYRY MLGQRKRALS KSKRE----- --
 PHS1   IPGFPMLFLH MVAQRKKVMK SLRSSFGKKL I-
```

S2 Fig
