# Supplementary material for: Dual Fatty Acid Elongase Complex Interactions in Arabidopsis
Source: PLoS One. 2016 Sep 1;11(9):e0160631. doi: 10.1371/journal.pone.0160631 (PMC5008698; doi:10.1371/journal.pone.0160631)
Supplement: S1 Table — Heterozygotes pas2-1/+ mutants transformed with different constructs with PTPLA or PAS2 coding sequence under the control of PAS2 or 35S promoters. Segregation was analyzed in T2 progeny of several independent transgenic lines (column 2) and only those segregating pas2-1 mutants were kept for analysis. Complementation was scored positive (column 3) when 25% segregating seedlings showed pas2-1 phenotype (column 4). The constructs expressing PAS2 with or without the GFP under its own promoter were used as control lines. Homozygote pas2-1/pas2-1 complemented lines showed wild-type phenotype (data not shown) and were identified by the bstnI CAPS marker (G622A substitution in the coding sequence). (PDF) [file pone.0160631.s012.pdf]

S1Table: Segregation analysis of transgenic lines expressing *PTPLA* or *PAS2*. Heterozygotes *pas2-1/+* mutants transformed with different constructs with *PTPLA* or *PAS2* coding sequence under the control of *PAS2* or 35S promoters. Segregation was analyzed in T2 progeny of several independent transgenic lines (column 2) and only those segregating *pas2-1* mutants were kept for analysis. Complementation was scored positive (column 3) when 25% segregating seedlings showed *pas2-1* phenotype (column 4). The constructs expressing *PAS2* with or without the *GFP* under its own promoter were used as control lines. Homozygote *pas2-1/pas2-1* complemented lines showed wild-type phenotype (data not shown) and were identified by the *bstnI* CAPS marker (G622A substitution in the coding sequence).

T2	Nb. *pas2/+* lines analized	Nb. complemented lines	*pas2-1* segregation
35S:GFP-PTPLA	3	0	25%
35S:PTPLA	2	0	25%
pPAS2:GFP-PTPLA	10	0	25%
pPAS2:PTPLA	14	0	25%
pPAS2:PTPLA-GFP	3	0	25%
pPAS2:GFP-PAS2	4	4	0
pPAS2:PAS2	7	4	0
pPAS2:PAS2-GFP	3	3	0
