# Supplementary material for: Dual Fatty Acid Elongase Complex Interactions in Arabidopsis
Source: PLoS One. 2016 Sep 1;11(9):e0160631. doi: 10.1371/journal.pone.0160631 (PMC5008698; doi:10.1371/journal.pone.0160631)
Supplement: S2 Table — The sequences in bold are not specific to the target gene. (PDF) [file pone.0160631.s013.pdf]

S2Table: Primers used for PCR cloning and quantitative PCR analysis. The sequences in bold are not specific to the target gene.

code	sens	target	Sequence
CM02	Forward	AT5G59770	5' GGGG ACA AGT TTG TAC AAA AAA GCA GGC TTC ATGTCGCCGTTTGTGAAGTT 3'
CM03	Reverse	AT5G59770	5' GGG GAC CAC TTT GTA CAA GAA AGC TGG GTC TCACATTCTCTTTCTCTTGC 3'
CM04	Reverse	AT5G59770	5' GGG GAC CAC TTT GTA CAA GAA AGC TGG GTC CATTCTCTTTCTCTTGCCGT 3'
CM14	Forward	AT5G59770	5' GCCGTGGCTGTCTATAACACTTG 3'
CM22	Forward	YJL097W/PHS1	5' ACTGCAGAA CCA GTGTGA TGG ATGTCAAAAAAACTTGCGT 3'
CM23	Reverse	YJL097W/PHS1	5' ACTGCAGAA CCA GTGTGC TGG TCAAATTAGTTTCTTCCCGA 3'
CM35	Forward	AT5G59770	5' TGTCGCCGTTTGTGAAGTTT 3'
CM36	Reverse	AT5G59770	5' CCAGCAGAAGCGTAAGCAGA 3'
LB1.3	Reverse	T-DNA	5' ATTTTGCCGATTTCGGAAC 3'
LG103	Reverse	AT5G59770	5' GCTTGGTGTATCGGTGAGGT 3'
LG116	Forward	AT5G10480	5' ACCCAAGCTTGGGCGGAATATCCACTGTTAGCT 3'
LG117	Reverse	AT5G10480	5' AGACTAGTCGGAAAGTGAATACGCGAGAA 3'
LG123	Forward	AT5G59770	5' ACGAGCTCGTCTAGGATTTGATTCATTTG 3'
LG124	Reverse	AT5G59770	5' AGACTAGTCGAGTCACTCGTACAGTAGTA 3'
LG73	Forward	YEL021W/URA3	5' CCCAACTGCACAGAACAAAAACCTGCAGGAAACGAAGATAAATCATGCGTACGCTGCAGGTCGAC 3'
LG74	Reverse	YEL021W/URA3	5' CCACGGTTCTATACTGTTGACCCAATGCGTCTCCCTTGTCATCTTTAATCGATGAATTCGAGCTCG 3'
LG79	Forward	AT5G59770	5' CCGTGAAGCTTCTTCGATTT 3'
LG94	Forward	AT5G59770	5' GGGGACAAGTTTGTACAAAAAAGCAGGCT TCTAGGATTTGATTCATTTG 3'
LG96	Reverse	AT5G59770	5' GGGGACCACTTTGTACAAGAAAGCTGGGT GAGTCACTCGTACAGTAGTA 3'
PAS2	Forward	AT5G10480	5' CGG AGA TGT ACA GCG TTA GGA TG 3'
PAS2	Reverse	AT5G10480	5' CAT GTG TGG GCT ACC TGG GAC 3'
PAS2 Q-PCR1 F	Forward	AT5G10480	5' CGGAGATGTACAGCGTTAGGATG 3'
PAS2 Q-PCR1 R	Reverse	AT5G10480	5' CATGTGTGGGCTACCTGGGAC 3'
Pas2_1F	Forward	AT5G10480	5' TAAAGCCATTTCCCTCAGACTC 3'
Pas2_1R	Reverse	AT5G10480	5' CTTTCGCTGACCAAGCATGTACC 3'
/	Forward	AT2G28390	5' AACTCTATGCAGCATTTGATCCACT 3'
/	Reverse	AT2G28390	5' TGATTGCATATCTTTATCGCCATC 3'
